# Broadband All‐Optical Memtransistor Based on Organic Cocrystals for Noise‐Robust Motion Recognition

**DOI:** 10.1002/advs.202515087

**Published:** 2025-12-02

**Authors:** Zhaohui Cai, Yuxiao Fang, Wenjie Du, Zhengjun Liu, Yingli Shi, Jiahua Luo, Rui Wu, Lixing Kang, Chun Zhao

**Affiliations:** ^1^ School of Advanced Technology Xi'an Jiaotong‐Liverpool University Suzhou 215123 P. R. China; ^2^ Suzhou Laboratory Suzhou 215000 P. R. China; ^3^ Department of Electrical Engineering and Electronics University of Liverpool Liverpool L69 3BX UK; ^4^ Suzhou Institute for Advanced Research University of Science and Technology of China Suzhou 215123 P. R. China; ^5^ School of Nano‐Tech and Nano‐Bionics University of Science and Technology of China Hefei 230026 P. R. China; ^6^ Advanced Materials Division Suzhou Institute of Nano‐Tech and Nano‐Bionics Chinese Academy of Sciences Suzhou 215123 P. R. China

**Keywords:** action recognition, all‐optical synaptic memtransistor, linearity control, neuromorphic vision system, optoelectronic modulation, organic charge transfer cocrystals

## Abstract

All‐optical artificial synaptic devices offer promising potential for neuromorphic computing, yet their development is hindered by limited spectral tunability and poor plasticity linearity. Here, a broadband all‐optical synaptic memtransistor based on organic charge transfer cocrystals (DTT‐TCNQ) is reported, which enables fully light‐driven and reversible modulation of synaptic weights across a wide wavelength range (395–808 nm). The device exhibits bidirectional excitatory and inhibitory photoresponses, and achieves highly linear long‐term potentiation and depression (LTP/LTD) characteristics with ultralow nonlinearity (α_p_ = 0.00191, α_d_ = 0.00305) and asymmetry ratio (AR = 0.00114), attributed to a synergistic strategy combining frequency modulation and photoelectric coupling. When integrated into a convolutional neural long short‐term memory network (CNN‐LSTM) hybrid network, the device enables rapid convergence (98.77% accuracy in 6 training epochs) and robust recognition performance under spatiotemporal noise, outperforming conventional light‐write/electric‐erasing schemes. This work bridges material‐level innovation and system‐level functionality, offering a scalable approach toward energy‐efficient, noise‐resilient neuromorphic vision systems.

## Graphical abstract

This work presents an all‐optical synaptic transistor using DTT‐TCNQ organic charge transfer cocrystals, achieving broadband bidirectional modulation (395–808 nm) with highly linear synaptic plasticity (nonlinearity coefficients α_p_ = 0.00191, α_d_ = 0.00305). The device demonstrates robust neuromorphic computing performance, reaching 98.77% accuracy within 6 training epochs in CNN‐LSTM networks for dynamic vision recognition.

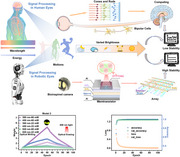

## 1 Introduction

Among various neuromorphic devices, all‐optical memtransistors represent a promising class of neuromorphic computing elements that operate exclusively under optical input. By mimicking the information processing mechanisms between the retina and the brain, these devices offer a unified platform for perception, signal processing, and memory storage, which is particularly attractive for intelligent vision systems.^[^ ^1^, ^2^, ^3^, ^4^ ^]^ Their functional mechanisms are rooted in photogenerated carrier dynamics‐where photoexcitation creates internal electric fields by trapping electron‐hole pairs at heterojunctions or defects‐and oxygen vacancy‐related photo‐redox reactions in oxide semiconductors, which modulate electron transport pathways through ionization state switching.^[^ ^5^, ^6^ ^]^ These devices enable high‐speed response, electromagnetic interference immunity, and energy‐efficient operations, which are critical for robust performance in complex optical environments.^[^ ^7^, ^8^ ^]^

Despite rapid progress, the advancement of high‐performance all‐optical synaptic devices is constrained by two fundamental challenges. First, most reported devices exhibit narrow spectral response ranges, typically confined to UV or visible bands (e.g., Si NCs/P3HT hybrid structures and SnSe‐based devices only demonstrate wavelength‐selective photoresponse below 600 nm), limiting their adaptability in real‐world multispectral environments.^[^ ^9^, ^10^ ^]^ Some efforts have explored wide‐bandgap semiconductors or 2D heterostructures to extend photoresponse coverage,^[^ ^11^ ^]^ yet robust bidirectional modulation under NIR illumination remains elusive.

Second, achieving highly linear and symmetric synaptic plasticity, especially in long‐term potentiation (LTP)/long‐term depression (LTD) modulation, remains difficult. This is mainly due to the complex interplay between photogenerated carriers, trap states, and interface dynamics, which often leads to nonlinear weight updates and asymmetric conductance changes.^[^ ^12^, ^13^, ^14^ ^]^ To compensate for limited modulation capability, many reported systems adopt hybrid control strategies, such as light‐write/electric‐erase schemes. However, such approaches introduce additional circuit complexity and consume more power, and often yield poor weight update linearity, particularly in the depression phase.^[^ ^15^, ^16^ ^]^ Moreover, few studies have realized reliable all‐optical LTD in the NIR range, where reduced photon energy makes effective modulation of trap states and vacancies highly challenging.^[^ ^17^, ^18^, ^19^ ^]^

To address these limitations, organic charge transfer cocrystals (OCTCs) offer a promising material platform. Comprising precisely stacked donor‐acceptor molecular units, OCTCs feature tunable energy levels, directional intermolecular interactions, and extended optical absorption due to charge‐transfer states.^[^ ^20^, ^21^, ^22^ ^]^ Their unique ability to simultaneously support broadband absorption and high mobility charge transport makes them ideal for constructing light‐driven, plasticity‐controllable neuromorphic transistors.^[^ ^23^ ^]^ Specifically, the DTT‐TCNQ cocrystal exhibits strong waveguiding effects and directionally aligned charge transport, enabling efficient carrier injection and extraction under both UVvis and NIR light. These features allow for precise modulation of synaptic states using purely optical inputs.

While recent advances have explored various material platforms, including carbon‐based systems achieving 97.3% recognition accuracy, perovskite devices with excellent photoresponse but stability issues, and 2D heterostructures with wavelength‐selective responses typically below 700 nm‐these approaches face fundamental constraints, most require hybrid electrical‐optical control for bidirectional modulation, and none have demonstrated effective LTD under near‐infrared illumination.^[^ ^24^, ^25^, ^26^, ^27^ ^]^ In this study, we demonstrate a fully all‐optical synaptic memtransistor based on DTT‐TCNQ cocrystal microsheets, capable of broadband, bidirectional plasticity modulation across 395–808 nm, including both LTP and near‐infrared‐triggered LTD. Unlike previous hybrid approaches, the device can achieve complete bidirectional plasticity modulation (both LTP and LTD) using only optical stimuli across an unprecedented broadband range (395–808 nm), specifically including near‐infrared LTD capability that has remained elusive. Most significantly, a frequency‐tuned optoelectronic synergy strategy is introduced to achieve nonlinearity coefficients as low as α_p_ = 0.00191 and α_d_ = 0.00305 with an asymmetry ratio of just 0.00114. When implemented in a convolutional neural long short‐term memory network (CNN‐LSTM) hybrid network, our all‐optical approach demonstrates superior noise tolerance and enables rapid convergence—reaching 98.77% accuracy within only 6 epochs, significantly faster than conventional approaches. This work establishes a complete material‐device‐system pathway that uniquely combines broadband all‐optical operation, near‐infrared LTD capability, and exceptional linearity, advancing toward truly robust, energy‐efficient neuromorphic vision hardware without electrical control complexity.

## 2 Results and Discussion

### 2.1 Biomimetic Architecture for All‐Optical Visual Systems

**Figure 1:**
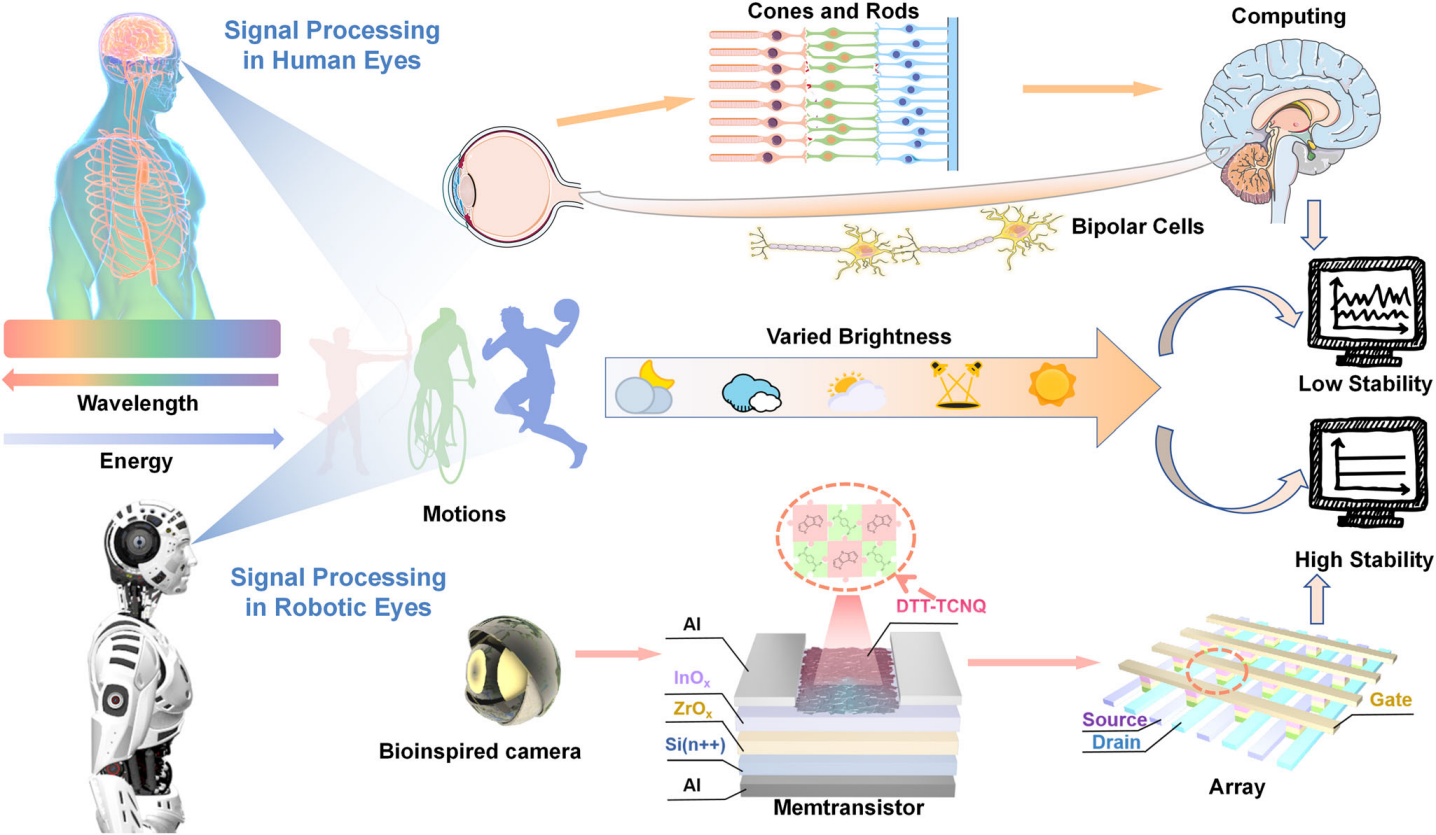
Signal Processing in Human and Robotic Vision Systems. The upper part illustrates human vision: Light signals are captured by cone cells (color detection) and rod cells (low‐light sensitivity), converted to electrical signals via photoreceptor transduction, processed by bipolar cells for contrast enhancement and signal conditioning, then transmitted to the visual cortex for pattern recognition and decision making. The lower part depicts robotic vision: broadband light (395–808 nm) is captured by the DTT‐TCNQ enhanced memtransistor array, where the InO_x_ layer performs photoelectric conversion, ZrO_x_ provides signal modulation through charge trapping/detrapping, and DTT‐TCNQ enables wavelength‐dependent synaptic plasticity. The arrayed circuit performs parallel processing analogous to retinal processing, outputting to neuromorphic computing systems for motion recognition.

Throughout biological evolution, the human visual system has developed a highly efficient signal processing mechanism. As shown in **Figure** **1**, its layered architecture enables precise light capture and seamless information processing from perception to cognition. The retina contains cone cells for bright‐light vision and rod cells for low‐light vision. Cone cells decode spectral information, while rod cells maintain visual function in dim conditions.^[^ ^28^, ^29^ ^]^ Bipolar cells transmit and encode light intensity variations, providing structured input for advanced visual processing in the cerebral cortex.^[^ ^30^ ^]^ However, traditional artificial vision systems often struggle with signal instability and poor adaptability under varying lighting conditions.^[^ ^31^ ^]^ Inspired by this, a DTT‐TCNQ cocrystal‐based all‐optical biomimetic visual system has been designed, emulating the retina's multilayer structure. The InO_x_/ZrO_x_/Si(n^++^) thin‐film structure mimics biological layers: InO_x_ handles photoelectric conversion, ZrO_x_ modulates signals, and the DTT‐TCNQ layer enhances optical waveguiding and environmental adaptability.^[^ ^32^ ^]^ This architecture ensures stable performance under fluctuating light conditions. In practical applications, the system excels in dynamic environments, such as tracking athletes in competitive sports, effectively filtering background noise and capturing motion details. Integrated with advanced neuromorphic computing, it enables real‐time image analysis and supports post‐game performance evaluation. This breakthrough offers promising solutions for next‐generation intelligent robotic vision systems and innovations in sports science.

### 2.2 Device Structure and Basic Characteristics

**Figure 2:**
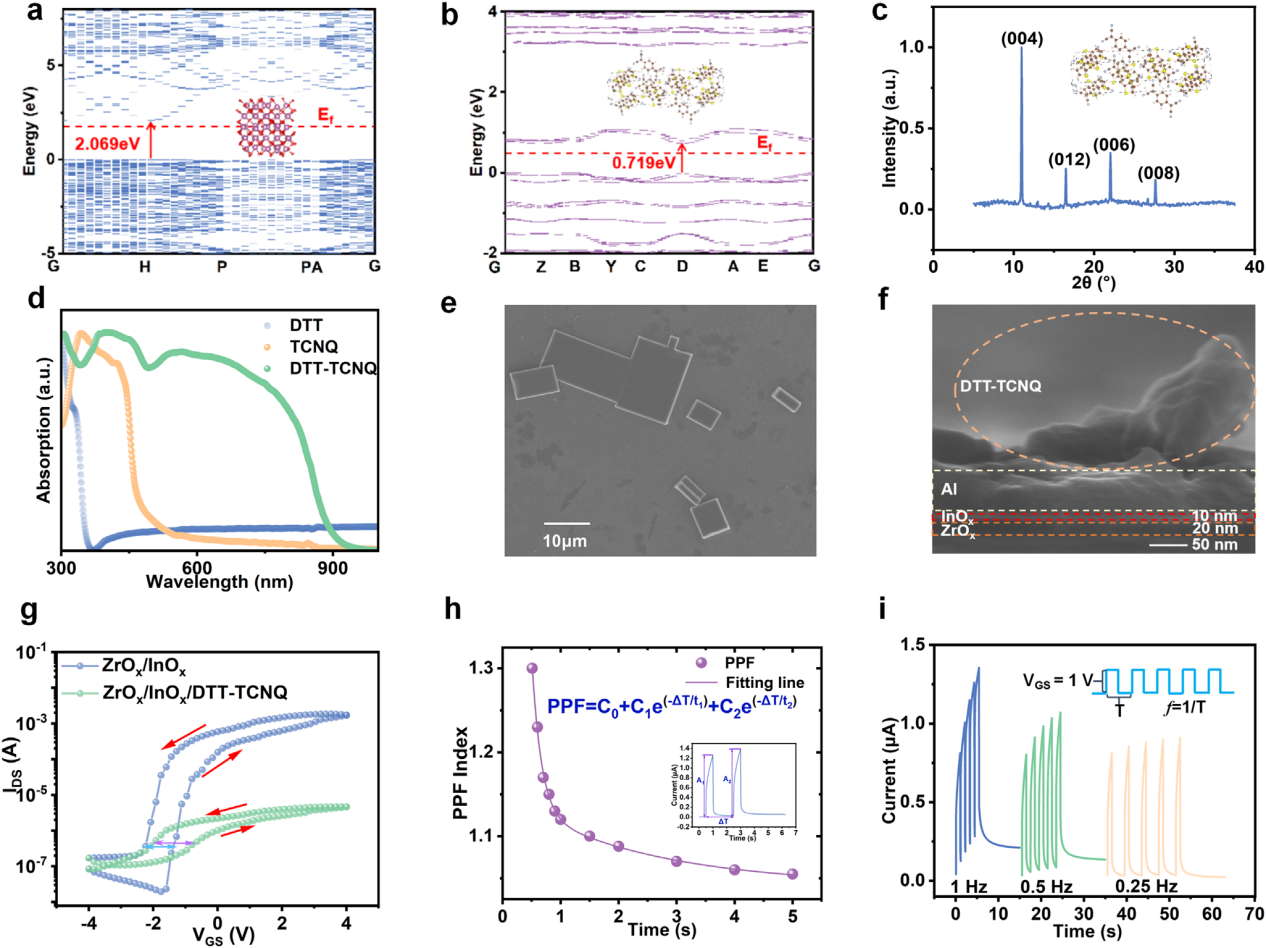
Characterization and electrical excitation characteristics of the DTT‐TCNQ enhanced memtransistor. a) First‐principles calculations of the band structure and Fermi level of InO_x_ with oxygen vacancies. b) Band structure and Fermi level of DTT‐TCNQ. c) XRD pattern of DTT‐TCNQ, showing characteristic diffraction peaks. d) UV–vis absorption spectra of DTT, TCNQ, and DTT‐TCNQ. e) SEM image of DTT‐TCNQ micro‐sheets on the substrate. f) Cross‐sectional SEM image of the memtransistor structure with distinct layers of DTT‐TCNQ, InO_x_, and ZrO_x_. g) Transfer characteristics (I_DS_‐V_GS_) of devices with and without DTT‐TCNQ layers. h) PPF index and its exponential fitting curve, highlighting short‐term synaptic plasticity. i) Frequency‐dependent EPSC of the memtransistor under varying electrical pulse frequencies.

Following the elucidation of the complex architecture and functionality of robotic vision systems, our research is focused on examining the material properties and the optoelectronic characteristics of the all‐optical control device, as these features establish the foundation for subsequent neural network design. Figure **2**a presents the band structure of InO_x_ with oxygen vacancies calculated through density functional theory (DFT). This configuration exhibits a calculated bandgap of 2.069 eV with the Fermi level positioned at 1.862 eV. Compared to ideal InO_x_ structure, oxygen vacancies reduce the bandgap by approximately1 eV and shift the Fermi level closer to the conduction band minimum (CBM).^[^ ^33^ ^]^ The electron distribution patterns within the InO_x_ structure, shown in Figure S1 (Supporting Information), reveal heterogeneity induced by oxygen vacancies, which fundamentally determines the electrical properties of InO_x_. The density of states (DOS) plot in Figure S2 (Supporting Information) demonstrates continuous state density near the Fermi level, indicating semiconductor characteristics and favorable carrier transport properties.^[^ ^34^ ^]^ DTT‐TCNQ exhibits a narrow bandgap of 0.719 eV with the Fermi level at 0.488 eV (Figure 2). As illustrated in Figures S3 and S4 (Supporting Information), DTT‐TCNQ forms distinct rectangular crystalline plates through self‐assembly driven by charge transfer interactions between DTT (donor) and TCNQ (acceptor).^[^ ^35^ ^]^ This cocrystal displays a unique biaxial molecular stacking pattern. The periodic charge density variations observed along the molecular stacking direction reveal strong intermolecular interactions facilitating directional charge transport. The DOS results for DTT‐TCNQ (Figure S5, Supporting Information) show no distinct energy gap near the Fermi level, favoring carrier transport. The pronounced oscillations and peaks observed in the −15 to −5 eV range originate from the quasi‐discrete density of states characteristics of the DTT and TCNQ molecular orbitals. Due to the quasi‐1D *π–π* stacking structure adopted by the molecules and the relatively weak intermolecular coupling (primarily van der Waals interactions), this results in limited band dispersion and retention of molecular orbital characteristics, thereby generating characteristic intramolecular *π–π* ^*^ transitions and intermolecular charge transfer optical absorption features.^[^ ^18^ ^]^ X‐ray diffraction (XRD) characterization (Figure 2c) reveals prominent characteristic peaks indicating highly crystalline DTT‐TCNQ microsheets. These peaks at 10.97°, 16.48°, 22.04°, and 22.60° correspond to the (004), (012), (006), and (008) crystal planes, respectively. The predominance of (00l) family reflections [(004), (006), (008)] indicates a strong preferred orientation with directional growth along the *c*‐axis direction.^[^ ^36^ ^]^ UV–vis spectroscopy analysis results (Figure 2d) demonstrate that the complex exhibits a maximum near‐infrared (NIR) emission peak at 860 nm, significantly red‐shifted compared to individual DTT and TCNQ crystals, a characteristic originating from intermolecular charge transfer (CT) interactions between donor and acceptor components. These CT interactions create new electronic transitions at lower energies than individual components, enabling electrons to transfer directly from the highest occupied molecular orbital (HOMO) of the donor to the lowest unoccupied molecular orbital (LUMO) of the acceptor, resulting in characteristic absorption bands in the visible to near‐infrared region.^[^ ^37^ ^]^ Simultaneously, the original electronic transitions of each component remain active, continuing to absorb in the ultraviolet to visible light range. This synergistic effect between localized electronic transitions and intermolecular CT transitions not only expands the material's optical response range but also enables all‐optical control characteristics and NIR‐induced excitation state transitions from MCT1 to MCT2.^[^ ^23^ ^]^ SEM imaging (Figure 2e) reveals cocrystal dimensions ranging from 10 to 110 µm. The basic device architecture (Figure 2f) comprises a ≈20 nm semiconductor layer, ≈10 nm dielectric layer, and a 100 nm metal electrode, with DTT‐TCNQ cocrystal thickness ranging from 100 to 300 nm when deposited on the top gate.

Electrical characterization reveals comprehensive performance features through output curves (I_DS_‐V_DS_) (Figure S9, Supporting Information). The device exhibits linear behavior at low drain voltage (V_DS_ = 0–0.5 V), transitioning to saturation at higher voltages. Increasing gate voltage (V_GS_) from 0 to 560 mV significantly enhances drain current, demonstrating excellent carrier concentration modulation capability. Figure 2g compares the transfer characteristics of the basic reference device and the device enhanced with DTT‐TCNQ microsheets. Under a fixed drain voltage of 1 V, the gate voltage has been swept from −4 to 4 V in the forward direction, and then from 4 to −4 V in the reverse direction. Both the original device and the modified device exhibit counterclockwise hysteresis windows, which is characteristic behavior of an *n*‐type device. This hysteresis effect primarily originates from charge trapping and release mechanisms within the dielectric layer, analogous to neurotransmitter transmission mechanisms in biological synapses.^[^ ^38^ ^]^ This demonstrates the device's capacity to emulate the learning and memory functions of biological synapses. The all‐optical device composed of DTT‐TCNQ exhibits a larger hysteresis window, indicating enhanced charge trapping and release responses. Figure 2h demonstrates that applying two consecutive identical gate pulses results in an enhanced current response to the second pulse (A_2_) compared to the first (A_1_). This enhancement occurs because the second pulse arrives while the tail post‐synaptic current (PSC) from the first pulse is still present. This phenomenon is a characteristic manifestation of short‐term plasticity, specifically paired‐pulse facilitation (PPF). The PPF index, a crucial parameter for quantifying the degree of synaptic enhancement, can be calculated as the ratio of the two peak current amplitudes:^[^ ^39^ ^]^

(1) $$PPF\;index=\frac{A_{2}}{A_{1}}\times 100\%$$

A_1_ and A_2_ represent the peak currents generated by the first and second pulse stimulations, respectively. The PPF index can also be fitted using the following exponential decay curve:

(2) $$PPF\;index=1+C_{1}e^{\left(-\frac{\Delta T}{\tau_{1}}\right)}+C_{2}e^{\left(-\frac{\Delta T}{\tau_{2}}\right)}$$

C_1_, and C_2_ refer to the initial facilitation amplitudes, ΔT denotes the time interval between two consecutive pulses, and τ_1_ and τ_2_ are the relaxation time constants. τ_1_ = 0.8 s and τ_2_ = 3.2 s, representing fast carrier recombination and slow trap state relaxation, respectively. The fast time constant represents rapid carrier recombination processes in the active layer, functioning analogously to the quick calcium clearance mechanisms observed in presynaptic terminals of biological synapses. The slow time constant is associated with gradual trap state relaxation and charge redistribution within the device structure, effectively mimicking the long‐lasting biochemical changes that occur in biological synapses.

As the interval between pulses (ΔT) increases, the PPF index decreases. From the inset of Figure 2h, it can be observed that when the pulse interval is 2 s, the PPF index is ≈110%. This decline in the PPF index with increasing ΔT reflects the memtransistors’ rapid response capability to sequential stimulations, showcasing the short‐term facilitation properties. As shown in Figure 2i, the peak currents exhibit frequency‐dependent characteristics. Under fixed experimental conditions (5 pulses, V_GS_ = 1 V, and 0.9 s pulse width), tests have been conducted at frequencies of 1, 0.5, and 0.25 Hz. Peak current shows a progressive increase as pulse frequency increases. Furthermore, a positive correlation is observed between pulse frequency and current retention duration following gate voltage removal. The postsynaptic response is also modulated by electrical pulse parameters, including voltage amplitude, pulse number, and pulse width (Figure S10, Supporting Information). Higher V_GS_ produces larger response currents, while lower V_GS_ demonstrates improved current retention ratio after the stimulus is removed. The excitatory postsynaptic current (EPSC) shows a saturation trend with increasing pulse numbers while maintaining a positive correlation between tail current intensity and pulse count. Additionally, doubling the pulse width from 0.4 to 1.6 s results in doubled peak current, demonstrating enhanced synaptic plasticity. These findings demonstrate the comprehensive dependence of synaptic response characteristics on multiple pulse parameters.

### 2.3 Wavelength‐Dependent Synaptic Response Analysis

**Figure 3:**
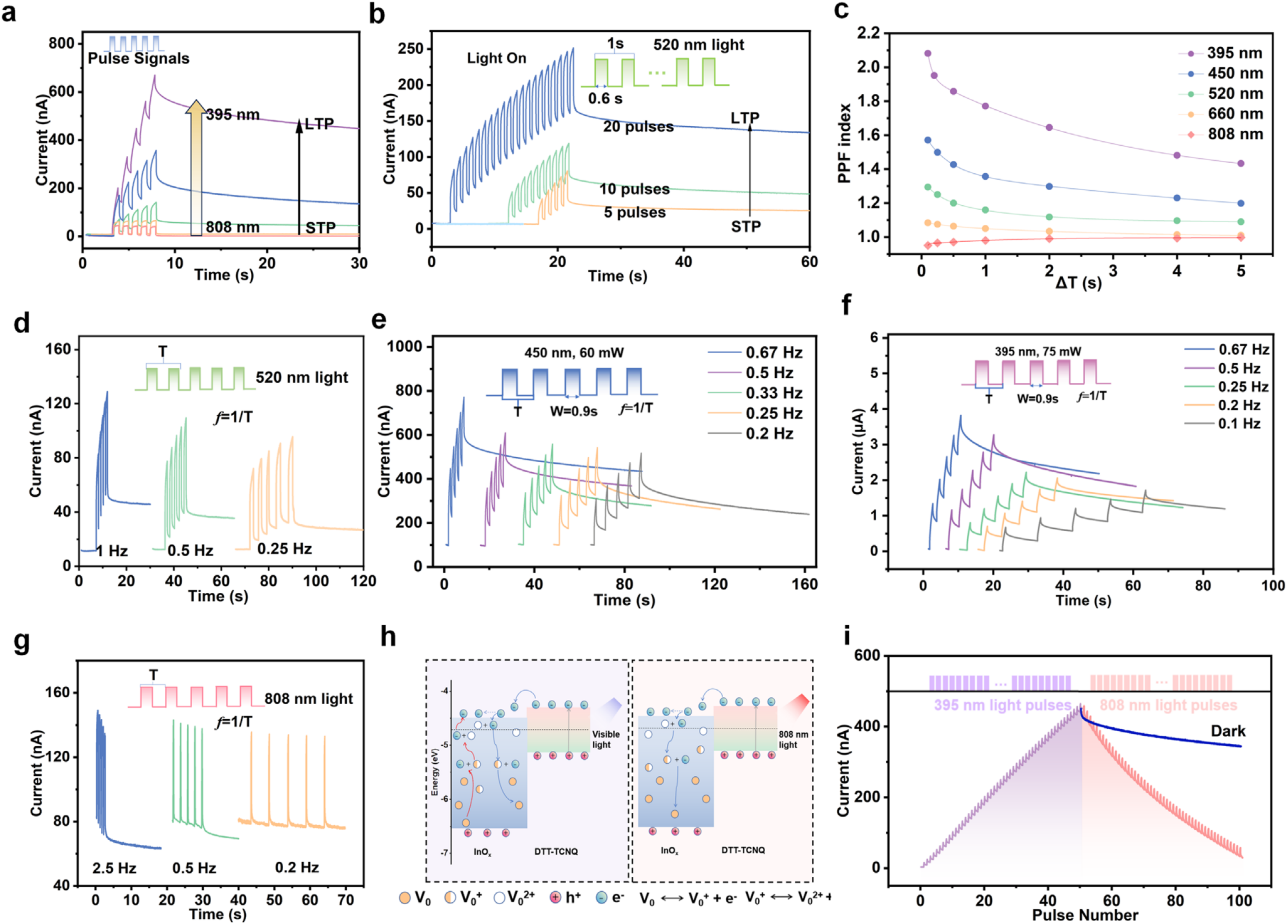
Photoexcitation characteristics of the DTT‐TCNQ enhanced memtransistor. a) EPSC under light pulses of varying wavelengths, demonstrating STP with 808 nm light to LTP with 395 nm light transition. b) Transition from STP to LTP with an increased number of 520 nm light pulses. c) Dynamic analysis of the PPF index over time across 395 to 808 nm. EPSC responses under d) 520 nm, e) 450 nm, and f) 395 nm light pulses. g) IPSC responses under varying frequencies of 808 nm light pulses. h) Schematic energy band diagrams of InO_x_ and DTT‐TCNQ before and after exposure of visible and 808 nm light, illustrating carrier transfer and vacancy dynamics. i) Optical LTP with 395 nm light and optical LTD with 808 nm light, highlighting reversible modulation of synaptic weights.

Under light stimulation across various wavelengths, the DTT‐TCNQ memtransistor shows enhanced LTP and synaptic plasticity compared to electrical stimulation due to its excellent optical waveguide properties. The devices demonstrate stable modulation and efficient learning under optical pulses, with particularly linear inhibition trends under infrared light, making it promising for neural network applications. The optical characteristics are studied by using multicolored light to mimic various cognitive learning and memory states typically observed in the human brain. The DTT‐TCNQ all optical devices have been subjected to five consecutive light pulses and illumination at different wavelengths (395, 450, 520, 660, and 808 nm, each at 40 mW power) under a bias of V_DS_ = 0.1 V. As shown in Figure **3**a, the devices exhibit wavelength‐dependent EPSC responses across the spectral range from ultraviolet to infrared, with higher currents observed at shorter wavelengths. Peak currents of 670, 356, 140, 63, and 41 nA are achieved under 395, 450, 520, 660, and 808 nm illumination, respectively. A transition from short‐term potentiation (STP) to LTP is observed as the light wavelength decreases. Notably, 395 nm UV light and 450 nm blue light exhibit high linearity, enabling more precise gradient weight adjustments in neural network training, facilitating convergence to desired solutions. The adaptability of EPSC is further validated under varying numbers of light pulses (Figure 3b), pulse power, and pulse width (Figure S11, Supporting Information). EPSC levels increase and approach saturation with increasing power and number of pulses, while extended pulse widths improve response linearity, enhancing stable weight updates in neural network training. Figure 3c presents the light‐induced PPF graph, showing distinct curve trends that vary with different wavelengths. The 395 nm UV exhibits the strongest PPF effect, with exponential values reaching above 2.0 (Figure S12 (Supporting Information) shows the PPF index for ultraviolet light at a double‐peak interval of 2 s). As wavelength increases, the PPF effect gradually weakens, with the PPF index falling below 1.0 at 808 nm (near‐infrared). Regarding temporal evolution characteristics, PPF curves from ultraviolet to red light all display similar decay trends, with varying decay rates under different wavelength excitations: shorter wavelengths exhibit more rapid decay, while longer wavelengths show relatively gradual decay. From an application viewpoint, this wavelength‐dependent PPF behavior establishes a foundation for controlling the weight linearity of all‐optical devices.

Based on the time‐dependent characteristics of artificial synapses, the nonlinearity of response current can be modulated by altering light pulse frequency, allowing flexible regulation of mapping weights in neuromorphic computing. Figure 3d–f illustrates EPSC variations with pulse frequency at 520, 450, and 395 nm, respectively. Higher pulse frequencies lead to higher peak current, larger tail current in LTP, and greater response linearity. Figure S13 (Supporting Information) shows that EPSC under 660 nm does not significantly increase with external stimulation. When the response current rises to a certain level and the light source is removed, introducing 808 nm infrared light accelerates the current decline. Figure 3g indicates that inhibitory postsynaptic current (IPSC) responses under infrared light decrease more with increasing pulse frequency. Figure S14 (Supporting Information) demonstrates the current response of the memtransistors during learning, forgetting, and relearning processes (450 nm, 40 mW). After five consecutive light pulses, EPSCs increase from 0.05 to 0.5 µA. Following a 5 s forgetting period, only two light pulses are required to restore the peak level of the first learning phase, with the second pulse group increasing EPSC from 0.25 to 0.63 µA. Compared to the first forgetting process, the tail current after the second phase increases by 0.22 µA. Research results indicate significantly enhanced current response after consecutive light pulses. Although current decays over time, it can quickly recover and strengthen upon re‐stimulation, demonstrating excellent relearning capability. This behavior, similar to human learning, is crucial for neuromorphic computing, effectively simulating synaptic plasticity in biological neurons.

The generation of IPSC can be understood by examining the vacancy structure and energy bands of the materials. As a typical n‐type oxide semiconductor, InO_x_ exhibits common oxygen vacancy defects, leading to persistent photoconductivity and electrical instability. Comparing the energy band alignments of InO_x_ (with oxygen vacancies) and DTT‐TCNQ (Figure S15, Supporting Information), the Fermi level of DTT‐TCNQ is further from its conduction band. When DTT‐TCNQ is grown on the InO_x_ thin film, electrons transfer from DTT‐TCNQ to InO_x_, forming an internal electric field directed from DTT‐TCNQ to InO_x_. As shown in Figure 3h, the InO_x_ thin film contains numerous ground‐state oxygen vacancies (V_o_) and ionized vacancies (V_o_^+^ and V_o_^2+^). The ionization of these vacancies releases free electrons, while the positively charged V_o_^+^ and V_o_^2+^ act as electron traps, reducing InO_x_ conductivity.^[^ ^40^ ^]^ Under visible light, both the InO_x_ channel and DTT‐TCNQ absorb light, activating ground‐state oxygen vacancies. Two competing processes affect channel conductivity: photogenerated electrons transfer from DTT‐TCNQ to InO_x_ to restore V_o_^+^ and V_o_^2+^, and the ionization of oxygen vacancies in InO_x_ dominates, producing more electrons. These results show a continuous increase in current under visible light stimulation. Infrared light specifically activates the cocrystal DTT‐TCNQ, generating photogenerated carriers that transfer to the InO_x_ channel, restoring V_o_^+^ and V_o_^2+^ to their ground state. This reduces InO_x_ channel conductivity, leading to negative photogenerated current. DTT‐TCNQ exhibits higher sensitivity to longer wavelengths, with the oxygen vacancy restoration process dominating under infrared light. Time‐resolved photoluminescence spectroscopy (TRPL) measurements in Figure S16 (Supporting Information) reveal systematic carrier lifetime variations from long‐lived DTT‐TCNQ to short‐lived InO_x_ with intermediate heterostructure lifetimes, demonstrating active electron transfer from DTT‐TCNQ to InO_x_ that directly supplies electrons for oxygen vacancy restoration, confirming that this lifetime reduction originates from interfacial charge transfer. Under 808 nm near‐infrared excitation, photogenerated electrons from DTT‐TCNQ transfer across the heterointerface to InO_x_ and recombine with ionized oxygen vacancies, leading to oxygen vacancy restoration that consumes free electrons and decreases the carrier concentration in the InO_x_ channel. The time‐resolved characteristics of TRPL data directly validate this electron transfer‐recombination mechanism as the fundamental physical origin of the observed inhibitory photoresponse.

This time‐resolved evidence quantitatively validates the oxygen vacancy restoration mechanism underlying the observed near‐infrared photoresponse behavior. As shown in Figure S17 (Supporting Information), external V_GS_ can modulate bidirectional light response. Under infrared light, applying positive V_GS_ facilitates electron transfer from DTT‐TCNQ to InO_x_, restoring most ionized oxygen vacancies and increasing ΔIPSC. Under visible light, positive gate voltage enhances ΔEPSC due to the combined activation of ground‐state oxygen vacancies by voltage and short‐wavelength light, producing excess electrons and higher response currents. Figure 3i illustrates LTD and LTP behaviors under light‐writing and light‐erasing stimulation. In the test, the devices exhibit LTP after 50 repeated light pulses (395 nm, 2 mW) and LTD after 20 repeated negative gate voltage pulses (808 nm, 55 mW). The infrared light‐induced ΔIPSC exceeds the value under dark conditions, achieving light‐writing and light‐erasing processes by adjusting infrared light frequencies, obtaining more highly linear and symmetric LTP/LTD curves compared to electrical writing and erasing (Figure S18, Supporting Information). The combination of highly linear and symmetric LTP and LTD behaviors enables efficient weight adjustment in neural networks, promoting dynamic learning and adaptability. Through continuous optimization of LTP and LTD control, these simulations improve neural network training efficiency, enhancing robustness and flexibility for complex tasks. In neural networks constructed from artificial synaptic devices, the linearity of weight updates is crucial. High linearity indicates that programmed conductance changes are proportional to the number of pulses, facilitating precise weight adjustments and improving learning outcomes. Furthermore, high linearity ensures predictable and stable programmed conductance changes, maintaining consistency throughout the training process in neural networks, accelerating convergence speed, and enhancing final performance. Figures S19 and S31 (Supporting Information) present the current cycling curves from a single device and the EPSC curves from multiple devices under 450 nm light, respectively. The memtransistor demonstrates cycle‐to‐cycle reliability, retaining 93.5% of its initial peak current (400 to 374.07 nA) after 15 repeated potentiation‐depression cycles, confirming robust long‐term operational stability for neuromorphic applications. Device‐to‐device testing of five independent devices shows high uniformity with a low coefficient of variation of 3.77%, indicating reliable fabrication reproducibility and consistent synaptic performance.

### 2.4 Memory and Logic Gate Performance

**Figure 4:**
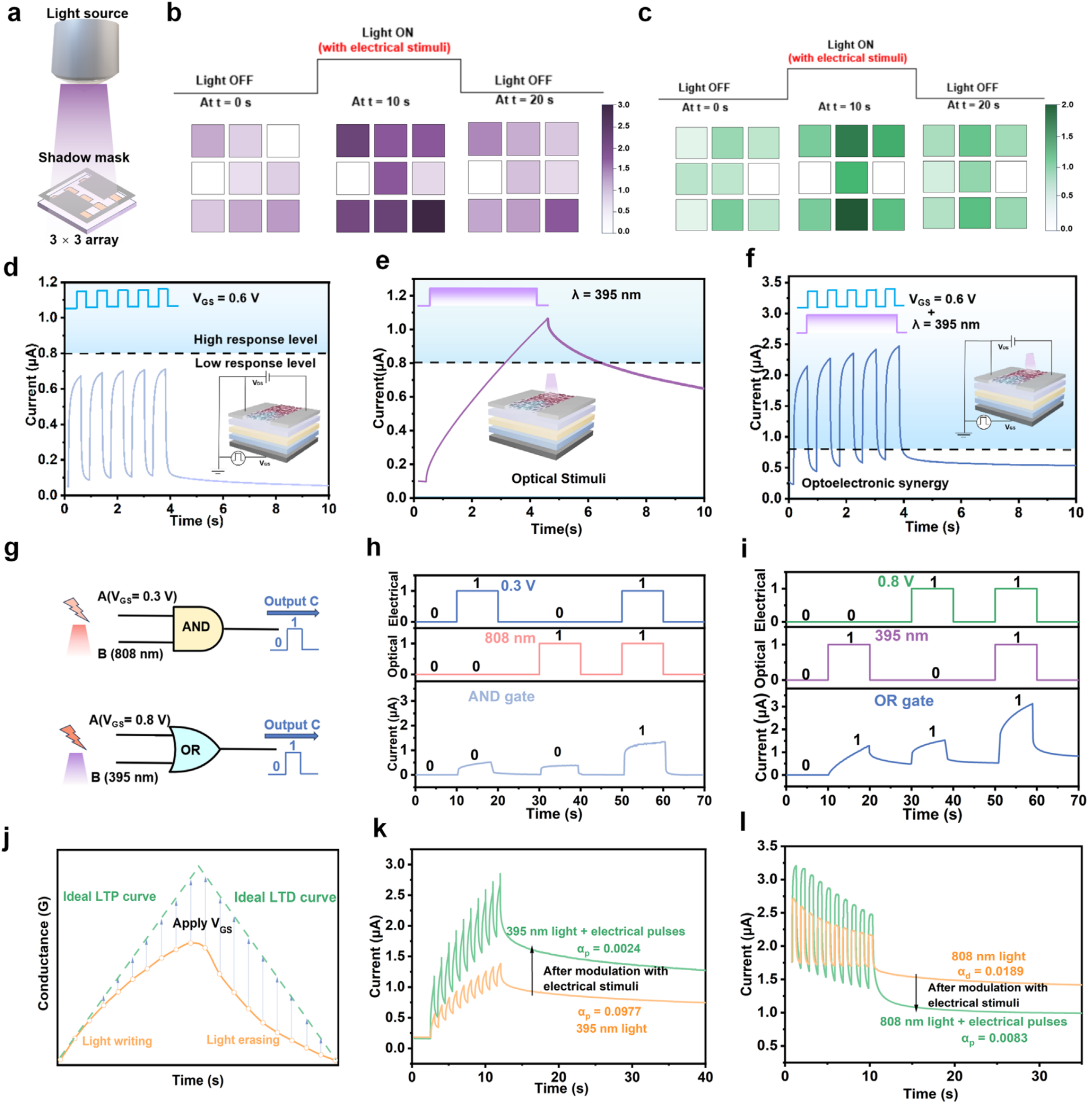
Optoelectronic responses and nonlinearity moderation of the DTT‐TCNQ enhanced memtransistor. a) Simulated demonstration of image processing for a 3 × 3 array based on experimentally measured single‐device characteristics, employing dark current‐limited noise model (SNR: 5271 dB) and device‐to‐device uniformity (coefficient of variation: 3.77%). b) under UV light illumination and c) under green light illumination. EPSC under d) gate voltage pulses (V_GS_ = 0.8 V), e) 395 nm light pulses, and f) combined electrical and optical pulses. g) Schematic diagrams of AND/OR logic gates integrated into the device. h) AND gate operation: electrical pulses (0.3 V) and 808 nm optical pulses as inputs, with output activated only when both inputs are present. i) OR gate operation: electrical pulses (0.8 V) and 395 nm optical pulses as inputs, with output triggered by either input. j) Optimization of LTP/LTD curves through optoelectronic modulation. Nonlinearity of k) EPSC under 395 nm light before and after electrical modulation, and l) IPSC under 808 nm light before and after electrical modulation.

The DTT‐TCNQ‐based devices exhibit high optical synaptic plasticity, enabling long‐term information retention akin to the biological brain. Inspired by the functionality of human eyes, Figure **4**a illustrates a schematic of an optical imaging setup for the letter “I” under applied gate voltage. The setup comprises a 3 × 3 array of DTT‐TCNQ all‐optical memtransistors, utilizing a shadow mask for letter imaging. During the imaging process, light of various colors passes through the mask featuring the letter I. Current from devices exposed to light is recorded after 10 s of illumination, generating a pixel image. The light is then turned off, and the current is recorded again after 20 s. Figure 4b,c displays the array pixel images under ultraviolet and green light, respectively. Initial current values recorded at t = 0 s are used for pixel images, as shown in the first column of Figure 4b,c. Compared to Figure S21 (Supporting Information) without electrical stimulation, the pixel images formed in the second column with electrical stimulation exhibit deeper colors, representing larger EPSC peaks. Under light stimulation, the devices exhibit significant LTP characteristics. This not only enables the array to maintain the previous states, but the tail currents also enhance the residual color intensities of the pixel images in the third column beyond their initial values. Figure 4d illustrates periodic current responses induced by voltage stimulation (V_GS_ = 0.6 V), simulating the formation of human STM. The transient response triggered by each voltage pulse mimics the temporary storage of perceptual information in working memory. At this stage, I_DS_ stays below the memory threshold (0.8 µA). The rapid decay of response intensity reflects the transient nature of short‐term memory, closely aligning with human processing of momentary information. Figure 4e depicts the progressive response curve induced by light stimulation (λ = 395 nm), corresponding to the dynamic process of memory consolidation. The all‐optical memtransistors exhibit higher I_DS_ values (1.05 µA), surpassing the memory threshold. The gradual rises in response intensity and subsequent plateau simulate the transition of information from short‐term to long‐term memory, while the slow decay in the later stage reflects the natural attenuation of memory strength over time. This dynamic characteristic bears similarity to hippocampus‐mediated memory enhancement processes.^[^ ^41^ ^]^ Figure 4f demonstrates the enhanced effect under photoelectric synergy, which illustrates the formation mechanism of associative memory. The superposition of photoelectric stimulation leads to a significant increase in response intensity, maintaining a higher level after the stimulation ends. This characteristic closely resembles the deep memory structure formed through multisensory inputs in humans, reflecting the multimodal integration characteristics of memory traces.

Figure 4g shows a schematic of the device used to record OR and AND logic gate outputs. Figure 4h demonstrates the physical realization of the AND gate. The system accepts two input signals: a 0.3 V electrical pulse and an 808 nm light pulse. This dual‐stimulation mode perfectly corresponds to the fundamental requirements of the logical “AND” operation at the physical level. Only when both voltage and light signals are present simultaneously (i.e., both inputs are “1”) does the output current reach a level above the threshold. Figure 4i illustrates the physical realization of the OR gate. The system similarly adopts a dual‐input approach: a 0.8 V voltage pulse and a 395 nm light pulse. In the physical mapping of OR logic, the presence of either input signal is sufficient to trigger an effective system response. This “compatibility” feature is manifested in the output current curve as a noticeable current response (>0.8 µA) to any non‐zero input combination (1–0, 0–1, 1–1). This response pattern precisely corresponds to the basic definition of logical “OR” operation, showcasing the flexibility of the physical system in implementing inclusive logical operations.

The synergistic combination of optical and electronic effects achieves two key benefits: it enables logic gate operations and allows for optical control of gate voltage. This dual functionality leads to improved linearity in both LTP and LTD characteristics. In Figure 4j, the tested light‐modulated curves (orange solid line) show a certain degree of non‐linear characteristics. By applying appropriate V_GS_, we can achieve precise control over conductance changes, thereby bringing the actual response closer to the ideal linear curve. This electric field‐assisted regulation strategy essentially modulates carrier transport behavior through an external field, with its principle illustrated in Figure S17 (Supporting Information). Figure 4k,l demonstrates the dynamic process of optoelectronic synergistic regulation under different wavelengths of light. The formula for calculating non‐linearity is:^[^ ^42^ ^]^

(3) $$G_{n+1}=G_{n}+\Delta G_{p}=G_{n}+\beta_{p}\exp\left(-\alpha_{p}\frac{G_{n}-G_{min}}{G_{max}-G_{min}}\right)$$

(4) $$G_{n+1}=G_{n}+\Delta G_{d}=G_{n}-\beta_{d}\exp\left(-\alpha_{d}\frac{G_{n}-G_{min}}{G_{max}-G_{min}}\right)$$

The Equation (3) is used for LTP, and Equation (4) is used for LTD, respectively. Where the G_n + 1_ and G_n_ represent the synaptic conductance of the device, and the parameters α and β are the step of change of nonlinearity and conductance, respectively. Under the synergistic effect of 395 nm light and electrical pulses, the system performs programmed conductance modulation, with the non‐linearity coefficient α_p_ decreasing from 0.0977 to 0.0024. Under 808 nm light conditions, through optoelectronic synergistic regulation, the system demonstrates superior linear response characteristics, with α_d_ reducing to 0.0083.

### 2.5 Neural Network Recognition Implementation

**Figure 5:**
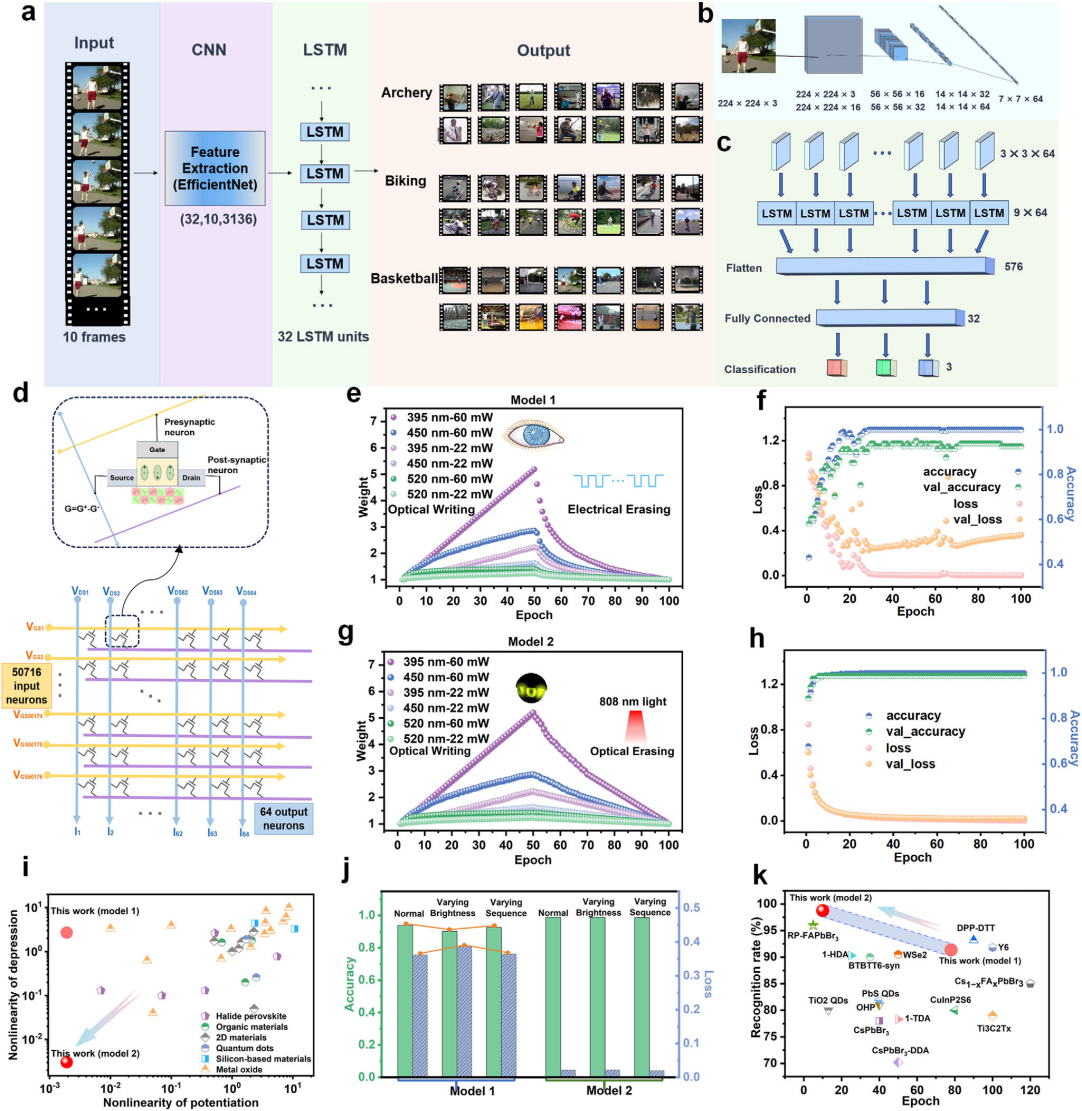
A neuromorphic computing system using the CNN‐LSTM algorithmic framework for the UCF‐101 video recognition task. a) Framework of the CNN‐LSTM model for recognizing motion categories (archery, biking, basketball) in video sequences. b) Structure of the CNN for feature extraction. c) Structure of the LSTM for temporal sequence processing. d) Transistor‐based array circuit for matrix operations, where V_GS_, V_DS_, and I represent the input signal, read signal, and output signal, respectively. e) Optical LTP and electrical LTD of synaptic weights over 100 epochs, simulating human visual recognition (Model 1). f) Recognition accuracy and loss for Model 1. g) Optical writing and optical erasing of synaptic weights over 100 epochs, leveraging 808 nm‐induced IPSC to simulate robotic visual recognition (Model 2). h) Recognition accuracy and loss for Model 2. i) Comparison of nonlinearity in potentiation and depression across various artificial synaptic devices (see references 1–35 in the Supporting Information). j) Comparison of loss and accuracy between Model 1 and Model 2 across normal recognition targets, targets with varying brightness, and targets with sequence variations. k) Noise interference performance: Comparison of this work's recognition accuracy and the number of epochs for stability with those reported in other studies.^[^ ^6^, ^13^, ^16^, ^40^, ^43^, ^44^, ^45^, ^46^, ^47^, ^48^, ^49^, ^50^ ^]^

Based on the aforementioned characteristics, a neuromorphic computing system is engineered utilizing DTT‐TCNQ memtransistors for human action recognition tasks. As illustrated in **Figure** **5**a, this system employs a hybrid convolutional neural‐long short‐term memory network (CNN‐LSTM) architecture, leveraging the stable performance of DTT‐TCNQ all‐optical devices under light stimulation. The architectural design involves preprocessing input videos into 10‐frame sequences, followed by spatial feature extraction using an EfficientNet‐based CNN, generating feature representations of dimension (32,10,3136) (Figure 5b). Subsequently, 32 LSTM units perform temporal modeling on these features, enabling cross‐frame temporal dependency analysis (Figure 5c). This network architecture adopts a transfer learning strategy, combining TimeDistributed layers to effectively capture spatiotemporal information in videos. At the same time, by freezing pre‐trained network layers, it reduces the number of trainable parameters, lowering the risk of overfitting. This allows the model to quickly converge to extremely high accuracy within very few training epochs. When facing high‐noise data, more linear weights often exhibit better generalization capabilities in the network, enabling the model to still converge to the same high recognition rate as in noise‐free environments. Based on the CNN‐LSTM hybrid neural network model illustrated in Figure S22 (Supporting Information), through three sets of convolution‐pooling combinations, the initial input features of dimension (10, 224, 224, 3) undergo progressive dimensionality reduction and feature extraction, ultimately transforming into 64 feature vectors at the CNN processing stage. The network architecture corresponds to the hardware implementation of a crossbar array comprising 50 716 input neurons and 64 output neurons, wherein each DTT‐TCNQ all‐optical control device serves as an artificial synaptic unit participating in matrix operations (Figure 5d). Through dimensional transformations (224 × 224 × 3→224 × 224 × 16→56 × 56 × 16→14 × 14 × 32→7 × 7 × 64) and multi‐layer LSTM processing (3 × 3 × 64), followed by 576‐dimensional feature flattening and a 32‐neuron fully connected layer, the system achieves accurate classification of action categories, including archery, cycling, and basketball.

Device performance is primarily constrained by key parameters such as the maximum/minimum conductance ratio (G_max_/G_min_), nonlinearity, and asymmetry of weight updates.^[^ ^43^, ^51^, ^52^, ^53^ ^]^ Addressing these factors, our study innovatively proposes and compares two regulatory strategies: a conventional light‐writing/electrical‐erasing scheme (Figure 5e, Model 1) and an all‐optical light‐writing/light‐erasing mechanism (Figure 5g, Model 2). Experimental results indicate that Model 2 achieves a high recognition accuracy of 98.77% within 6 epochs (Figure 5h), while Model 1 requires ≈100 training cycles to reach a recognition accuracy of 92.59% (Figure 5f). This excellent performance stems from the device's favorable LTP/LTD linearity and symmetry. As shown in Figure 5i, compared to various artificial synaptic devices reported in other literature, our work achieves superior linear control capability, with nonlinearity values of α_p_ = 0.00191 and α_d_ = 0.00305 calculated for Model 2 using Equations (3) and (4). Further analysis reveals that the device's asymmetry ratio AR (calculated as AR = |α_p_‐α_d_|) is merely 0.00114, significantly outperforming most artificial synaptic devices constructed from optoelectronic material systems (Figure S23, Supporting Information). The above results also show that compared to Model 1 (α_p_ = 0.00191, α_d_ = 2.6933, AR = 2.69139), Model 2 has better linearity and symmetry. To validate the reproducibility of these exceptional linearity characteristics, five independent devices were fabricated and tested under identical experimental conditions (optical LTP with 395 nm light at 40 mW and optical LTD with 808 nm light at 55 mW). Figure S20 (Supporting Information) presents the extracted normalized conductance curves from the five devices. These results demonstrate that our devices exhibit exceptional reliability essential for neural network simulations, featuring robust device‐to‐device consistency that fulfills the demanding requirements of neuromorphic computing applications.

To comprehensively evaluate system performance, detailed classification recognition performances are presented for both models through confusion matrices in Figure S24 (Supporting Information), with a more complete analysis of nonlinearity and asymmetry parameters systematically summarized in Table S1 (Supporting Information). These research findings conclusively validate the significant value of the proposed all‐optical control strategy in optimizing neuromorphic computing performance.

To assess the robustness of the developed neuromorphic computing system in complex environments, two typical noise interference tests have been specifically designed: brightness fluctuations caused by light intensity variations and information sequence disturbances resulting from temporal disorder. In practical application scenarios (Figure S25, Supporting Information), the system faces two primary challenges: First, dynamic changes in background light significantly affect the contrast between targets and backgrounds through pixel saturation (bright conditions) and photocurrent attenuation (dark conditions), leading to blurred object boundaries and increasing detection and recognition difficulties. Second, delays or disturbances in external signals cause shifts and disorder in time‐series data, posing challenges to accurate behavior and action recognition. Through performance evaluations of accuracy, loss, F1 score, and precision, we discovered significant differences in noise resistance between the two models. As shown in Figures S26–S30 (Supporting Information) and Figure 5j, the conventional light‐write/electrical‐erase scheme (Model 1) exhibits notable fluctuations in accuracy and loss under both types of noise interference. In contrast, Model 2, employing the light‐write/light‐erase mechanism, maintains performance levels comparable to noise‐free conditions even in high‐noise environments. Further comparative studies (Figure 5k) demonstrate that the all‐optical device based on DTT‐TCNQ exhibits exceptional noise resistance, achieving a stable recognition rate of 98.77% in noisy environments within just 10 iteration cycles (only requiring 4 more iteration cycles than in normal environments, significantly outperforming other artificial synaptic devices reported in the literature.^[^ ^6^, ^13^, ^16^, ^40^, ^43^, ^44^, ^45^, ^46^, ^47^, ^48^, ^49^, ^50^ ^]^ The light‐controlled write and erase mechanisms enable precise weight modulation, resulting in superior noise immunity. This stability allows the system to maintain accurate pattern recognition and decision‐making capabilities even in challenging environmental conditions. Beyond demonstrating the viability of DTT‐TCNQ‐based all‐optical synaptic memtransistors for neuromorphic computing, this work presents a novel approach to developing robust machine vision systems that can adapt to diverse environments.

## 3 Conclusion

This study reports notable advancements in the development of all‐optical memtransistors, supported by a carefully designed material and device framework. By leveraging DTT‐TCNQ microsheets, we developed broadband all‐optical pulse‐responsive devices with operational wavelengths spanning from 395 to 808 nm. Our approach, integrating precise light pulse parameter regulation with optoelectronic synergy, enabled the realization of LTP/LTD characteristics with low nonlinearity values (α_p_ = 0.00191, α_d_ = 0.00305) and an AR of just 0.00114. These results provide a foundation for efficient and accurate signal processing in neuromorphic systems. In practical applications, the robotic vision system constructed with the DTT‐TCNQ‐based all‐optical device demonstrated strong performance in dynamic and complex scenarios. Utilizing a CNN‐LSTM architecture, the system achieved a recognition accuracy of 98.77% on the UCF101 action recognition dataset within just 10 training cycles in noisy environments, exhibited notable interference resistance, maintaining high recognition accuracy, surpassing reported human‐level performance in motion recognition tasks. These features suggest that the system is capable of sustaining reliable operation under challenging conditions. The findings of this work contribute to advancing high‐performance, all‐optical neuromorphic computing devices and systems. Beyond device and material‐level innovations, this study offers a potential approach for achieving precise and robust signal modulation, providing a pathway for the development of intelligent vision systems with strong anti‐interference capabilities. As this technology is further refined and applied, it may hold promise for AI applications in complex and dynamic environments, paving the way for progress in human‐machine interaction and autonomous decision‐making.

## 4 Experimental Section

### Materials Preparation

The ZrO_x_ precursor solution was prepared by dissolving 7.5 mmol zirconium nitrate hydrate (Zr(NO_3_)_2_·xH_2_O) in 5 mL of deionized water. The mixture was stirred for 4 h to obtain a transparent solution. The InO_x_ precursor solution was prepared by dissolving 0.75 mmol indium nitrate hydrate (In(NO_3_)_2_·xH_2_O) in 5 mL of deionized water. The solution was stirred for 2 h to reach a completely dissolved state. The cocrystal material DTT‐TCNQ was synthesized using the solution‐evaporation method. DTT‐TCNQ was obtained by mixing 2.5 mmol DTT and 2.5 mmol TCNQ with 4 mL DCM and 2 mL ethanol. The solution mixture was shaked for 1 min and then was dropped onto a substrate. After 2 min, DTT‐TCNQ cocrystal microsheets were observed when the solvent evaporated completely.

### Device Fabrication

First, a heavily doped silicon substrate was cleaned with 30% concentrated hydrofluoric acid and deionized water, then was dried under a nitrogen flow. Next, the cleaned substrate was subjected to UV‐ozone treatment for 30 min to allow the film surface hydrophilic. The ZrO_x_ film was obtained by spin‐coating the previously prepared precursor solution at 4500 rpm for 30 s, followed by annealing in an air atmosphere at 300 °C for 2 h. The temperature was chosen to achieve optimal crystallization without degrading the oxide structure, while the duration ensures complete removal of residual nitrates and formation of stable Zr─O bonds. The InO_x_ film was obtained by spin‐coating the pre‐made precursor solution at 3000 rpm for 20 s and then was annealed in an air atmosphere at 200 °C for 1 h. This temperature range (180–220 °C) has been shown to optimize the balance between crystallinity and defect concentration in indium oxide thin films, ensuring both adequate structural order and the appropriate level of oxygen vacancies necessary for maintaining optimal electrical properties. Subsequently, 0.1 mL of the well‐mixed DTT‐TCNQ solution was drop‐coated onto the surface of the formed InO_x_ film. Finally, a 100 nm‐thick Al source‐drain electrode was fabricated onto the substrate through thermal evaporation using a shadow mask.

### Characterization

The X‐ray diffraction (XRD) data were tested by a D/max 2400 X‐ray diffractometer with Cu Kα radiation (λ = 1.54050 Å) measured in the 2θ range from 5° to 25°. A scanning electron microscope (SEM, SU8600) was performed to obtain the surface morphology of DTT‐TCNQ and the cross‐section morphology of the memtransistor. The light absorption peaks of DTT, TCNQ, and DTT‐TCNQ cocrystal were evaluated by using a UV–vis spectrophotometer (Perkin Elmer Lambda 750). The optoelectronic performances of the base transistor and the cocrystal‐enhanced transistor were examined in an enclosed chamber at room temperature using a semiconductor analyzer (Keysight, B1500 A). The analyzer is equipped with a probe station and laser diodes with wavelengths from 395 nm to 808 nm.

### Computational Methods

Density functional theory (DFT) calculations were conducted on a Hygon system equipped with two AMD 7742 64‐core processors using the Vienna Ab Initio Simulation Package (VASP). The Perdew–Burke–Ernzerhof (PBE) functional was employed within the generalized gradient approximation (GGA) to model electron exchange and correlation.^[^ ^54^, ^55^ ^]^ To describe the interaction between core and valence electrons, projector‐augmented plane wave (PAW) potentials were utilized, with valence electrons treated using a plane wave basis set.^[^ ^56^ ^]^ A kinetic energy cutoff of 500 eV was applied. For the Kohn–Sham orbitals, partial occupancies were permitted using the Gaussian smearing technique with a smearing width of 0.05 eV. Convergence of the electronic energy was achieved when the energy change was less than 10^−8^ eV. Geometry optimization was considered convergent when the Hellmann–Feynman forces were smaller than −0.02 eV Å^−1^. During the structural relaxation, the Brillouin zone was sampled using a Γ‐centered k‐point mesh of 3 × 3 × 3 for InO_x_, and 5 × 4 × 1 for DTT‐TCNQ. Spin polarization effects were included in all calculations.^[^ ^57^ ^]^

## Conflict of Interest

The authors declare no conflict of interest.

## Supporting information



Supporting Information

## Data Availability

The data that support the findings of this study are available from the corresponding author upon reasonable request.
