# Supplementary material for: Broadband All‐Optical Memtransistor Based on Organic Cocrystals for Noise‐Robust Motion Recognition
Source: Adv Sci (Weinh). 2025 Dec 2;13(8):e15087. doi: 10.1002/advs.202515087 (PMC12884737; doi:10.1002/advs.202515087)
Supplement: Supplementary file 1 — Supporting Information [file ADVS-13-e15087-s001.docx]

**Broadband All-Optical Memtransistor Based on Organic Cocrystals for Noise-Robust Motion Recognition**

*Zhaohui Cai^+,1,2^, Yuxiao Fang^+,1^**, Wenjie Du^+,4^, Zhengjun Liu^1,3^, Yingli Shi^1^, Jiahua Luo^1,3^, Wu Rui^1,3^,Lixing Kang^*,5,6^*, *Chun Zhao^*,1^*

1. School of Advanced Technology, Xi’an Jiaotong-Liverpool University, Suzhou 215123, P.R. China
2. Suzhou Laboratory, Suzhou 215000, China
3. Department of Electrical Engineering and Electronics, University of Liverpool, Liverpool L69 3BX, UK
4. Suzhou Institute for Advanced Research, University of Science and Technology of China, Suzhou, 215123, China
5. School of Nano-Tech and Nano-Bionics, University of Science and Technology of China, Hefei 230026, China
6. Advanced Materials Division, Suzhou Institute of Nano-Tech and Nano-Bionics, Chinese Academy of Sciences, Suzhou 215123, China

+ These authors contributes equally: Zhaohui Cai, Yuxiao Fang, Wenjie Du.

* Corresponding authors: [Chun.Zhao@xjtlu.edu.cn,](mailto:Chun.Zhao@xjtlu.edu.cn,) [lxkang2013@sinano.ac.cn](mailto:lxkang2013@sinano.ac.cn)

**Contents**

**[Figure S1](#_Figure S1. (a) The diagram of charge density for the optimal InOx structure with oxygen vacancies, where yellow represents accumulation of electrons. (b) The slice of charge densities in InOx . The red color represents higher electron density, while the blue color represents lower electron density.)**[. 1](#_Figure S1. (a) The diagram of charge density for the optimal InOx structure with oxygen vacancies, where yellow represents accumulation of electrons. (b) The slice of charge densities in InOx . The red color represents higher electron density, while the blue color represents lower electron density.)

**[Figure S2.](#_Figure S2. Calculated total DOS of InOx with O vacancies.)** [2](#_Figure S2. Calculated total DOS of InOx with O vacancies.)

**[Figure S3..](#_Figure S3. Model structures of (a) DTT, (b) TCNQ and (c) DTT-TCNQ.)** [3](#_Figure S3. Model structures of (a) DTT, (b) TCNQ and (c) DTT-TCNQ.)

**[Figure S4.](#_Figure S4. (a) The diagram of charge density for the optimal DTT-TCNQ structure, where yellow (blue) represents accumulation (depletion) of electrons. (b) The slice of charge densities in DTT-TCNQ. The red color represents higher electron density, while the blue color represents lower electron density.)** [4](#_Figure S4. (a) The diagram of charge density for the optimal DTT-TCNQ structure, where yellow (blue) represents accumulation (depletion) of electrons. (b) The slice of charge densities in DTT-TCNQ. The red color represents higher electron density, while the blue color represents lower electron density.)

**[Figure S5.](#_Figure S5. Calculated total DOS of DTT-TCNQ cocrystal.)** [5](#_Figure S5. Calculated total DOS of DTT-TCNQ cocrystal.)

**[Figure S6.](#_Figure S6. SEM image of a single DTT-TCNQ micro-sheet.)** [6](#_Figure S6. SEM image of a single DTT-TCNQ micro-sheet.)

**[Figure S7.](#_Figure S7. Image of DTT-TCNQ micro-sheets under microscope.)** [7](#_Figure S7. Image of DTT-TCNQ micro-sheets under microscope.)

**[Figure S8.](#_Figure S8. Side view of DTT-TCNQ micro-sheets under SEM.)** [8](#_Figure S8. Side view of DTT-TCNQ micro-sheets under SEM.)

**[Figure S9.](#_Figure S9. IDS-VDS curve of DTT-TCNQ enhanced memtransistor (VGS ranging from 0 mV to 540 mV).)** [9](#_Figure S9. IDS-VDS curve of DTT-TCNQ enhanced memtransistor (VGS ranging from 0 mV to 540 mV).)

**[Figure S10.](#_Figure S10. (a) EPSC and (b) IPSC stimulated by different VGS of electrical pulses (c) EPSC stimulated by different number, (d) widths of electrical pulses.)** [10](#_Figure S10. (a) EPSC and (b) IPSC stimulated by different VGS of electrical pulses (c) EPSC stimulated by different number, (d) widths of electrical pulses.)

**[Figure S11.](#_Figure S11. EPSC stimulated by (a) varying power of 520 nm light pulses with a width of 0.6 s, (b) varying width of 520 nm light pulses .)** [11](#_Figure S11. EPSC stimulated by (a) varying power of 520 nm light pulses with a width of 0.6 s, (b) varying width of 520 nm light pulses .)

**[Figure S12.](#_Figure S12. Two optical pulses of the same intensity (395 nm, 60 mW).)** [12](#_Figure S12. Two optical pulses of the same intensity (395 nm, 60 mW).)

**[Figure S13.](#_Figure S13. EPSC stimulated by varying frequencies of 660 nm optical pulses with a width of 0.9 s.)** [13](#_Figure S13. EPSC stimulated by varying frequencies of 660 nm optical pulses with a width of 0.9 s.)

**[Figure S14.](learning-forgetting-relearning#_Figure%20S14.%20Simulated%20)** [14](learning-forgetting-relearning#_Figure%20S14.%20Simulated%20)

**[Figure S15.](#_Figure S15. Schematic energy band diagrams of InOx and DTT-TCNQ before contact.)** [15](#_Figure S15. Schematic energy band diagrams of InOx and DTT-TCNQ before contact.)

**[Figure S16.](#_Figure S16. (a) Schematic illustration of energy band alignment for InOx/DTT-TCNQ heterojunction under NIR light stimulation (VGS > 0) and (b) under visible light stimulation (VGS > 0).)** [16](#_Figure S16. (a) Schematic illustration of energy band alignment for InOx/DTT-TCNQ heterojunction under NIR light stimulation (VGS > 0) and (b) under visible light stimulation (VGS > 0).)

**[Figure S17.](#_Figure S17. Writing characteristics under 395nm light pulses and erasing characteristics under negative electrical pulses (-1 V).)** [17](#_Figure S17. Writing characteristics under 395nm light pulses and erasing characteristics under negative electrical pulses (-1 V).)

**[Figure S18.](#_Figure S18. Pixel images generated by the array for the letter I under UV light (λ = 395 nm) and green light (λ = 520 nm).)** [18](#_Figure S18. Pixel images generated by the array for the letter I under UV light (λ = 395 nm) and green light (λ = 520 nm).)

**[Figure S19.](#_Figure S19. Time-Distributed CNN-LSTM Network Architectures.)** [19](#_Figure S19. Time-Distributed CNN-LSTM Network Architectures.)

**[Figure S20.](#_Figure S20. Comparison of the AR characteristics of among different types of synaptic devices.)** [20](#_Figure S20. Comparison of the AR characteristics of among different types of synaptic devices.)

**[Figure S21.](#_Figure S21. Confusion matrix containing the confidences of each class for (a) model 1 and (b) model 2.)** [21](#_Figure S21. Confusion matrix containing the confidences of each class for (a) model 1 and (b) model 2.)

**[Figure S22.](#_Figure S22. Video targets under noise of varying brightness and varying sequence.)** [22](#_Figure S22. Video targets under noise of varying brightness and varying sequence.)

**[Figure S23.](#_Figure S23. (a) Loss and (b) accuracy of model 1 after adding the noise of varying brightness.)** [23](#_Figure S23. (a) Loss and (b) accuracy of model 1 after adding the noise of varying brightness.)

**[Figure S24.](#_Figure S24. (a) Loss and (b) accuracy of model 2 after adding the noise of varying brightness.)** [24](#_Figure S24. (a) Loss and (b) accuracy of model 2 after adding the noise of varying brightness.)

**[Figure S25.](#_Figure S25. (a) Loss and (b) accuracy of model 1 after adding the noise of varying frame sequence.)** [25](#_Figure S25. (a) Loss and (b) accuracy of model 1 after adding the noise of varying frame sequence.)

**[Figure S26.](#_Figure S26. (a) Loss and (b) accuracy of model 2 after adding the noise of varying frame sequence.)** [26](#_Figure S26. (a) Loss and (b) accuracy of model 2 after adding the noise of varying frame sequence.)

**[Figure S27.](#_Figure S27. (a) F1 score and (b) precision of model 1 and model 2.)** [27](#_Figure S27. (a) F1 score and (b) precision of model 1 and model 2.)

**[Figure S28.](#_Figure S27. (a) F1 score and (b) precision of model 1 and model 2.)** [28](#_Figure S27. (a) F1 score and (b) precision of model 1 and model 2.)

**[Figure S29.](#_Figure S27. (a) F1 score and (b) precision of model 1 and model 2.)** [29](#_Figure S27. (a) F1 score and (b) precision of model 1 and model 2.)

**[Figure S30.](#_Figure S27. (a) F1 score and (b) precision of model 1 and model 2.)** [3](#_Figure S27. (a) F1 score and (b) precision of model 1 and model 2.)0

**[Figure S31.](#_Figure S27. (a) F1 score and (b) precision of model 1 and model 2.)** [3](#_Figure S27. (a) F1 score and (b) precision of model 1 and model 2.)1

**[Table S1.](#_Table S1. Comparison of the AR and NL characteristics of synaptic devices.)** [3](#_Table S1. Comparison of the AR and NL characteristics of synaptic devices.)2

**[References](#_References)** [33](#_References)

# **Figure S1.** (a) The diagram of charge density for the optimal InO_x_ structure with oxygen vacancies, where yellow represents accumulation of electrons. (b) The slice of charge densities in InO_x_ . The red color represents higher electron density, while the blue color represents lower electron density.


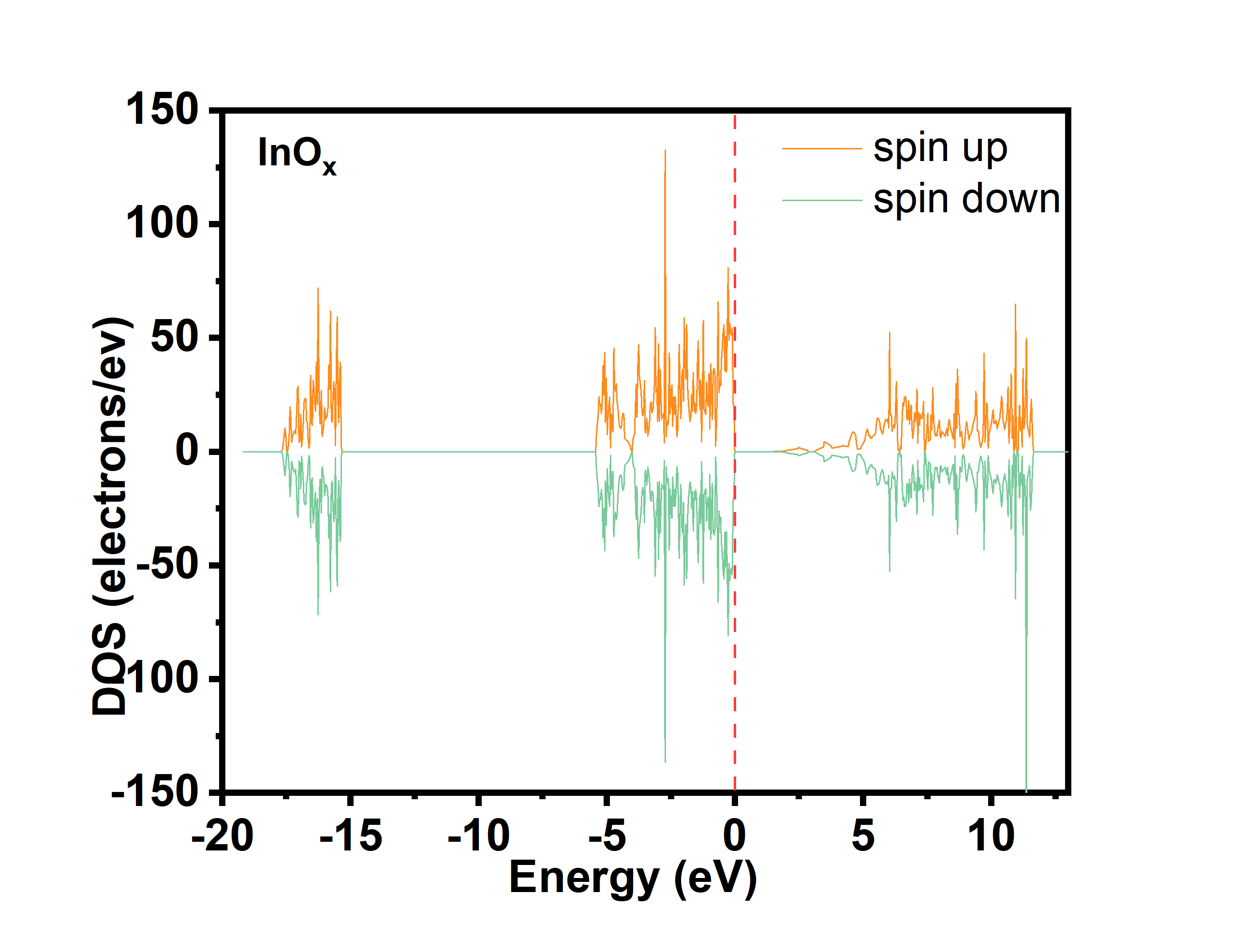


# **Figure S2.** Calculated total DOS of InO_x_ with O vacancies.

# Figure S3. Model structures of (a) DTT, (b) TCNQ and (c) DTT-TCNQ.

# Figure S4. (a) The diagram of charge density for the optimal DTT-TCNQ structure, where yellow (blue) represents accumulation (depletion) of electrons. (b) The slice of charge densities in DTT-TCNQ. The red color represents higher electron density, while the blue color represents lower electron density.


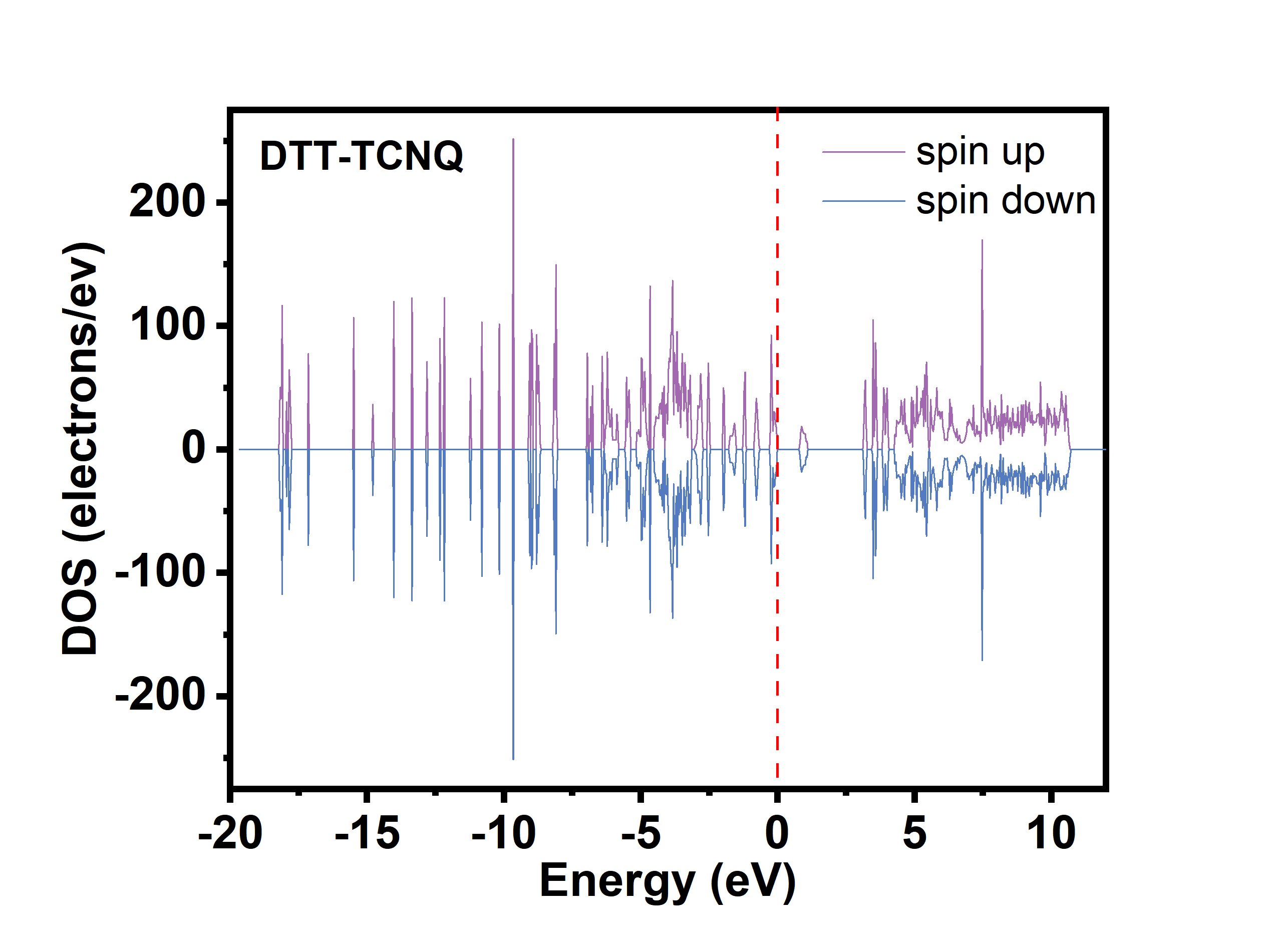


# Figure S5. Calculated total DOS of DTT-TCNQ cocrystal.


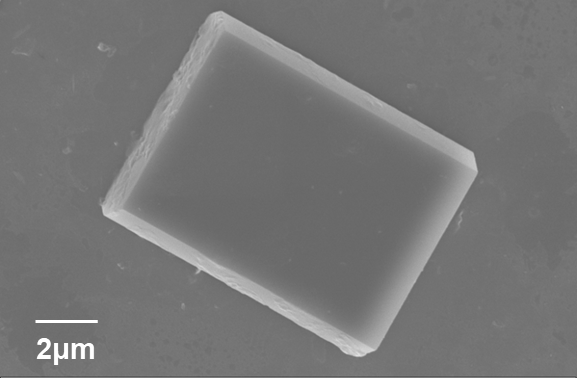


# Figure S6. SEM image of a single DTT-TCNQ micro-sheet.


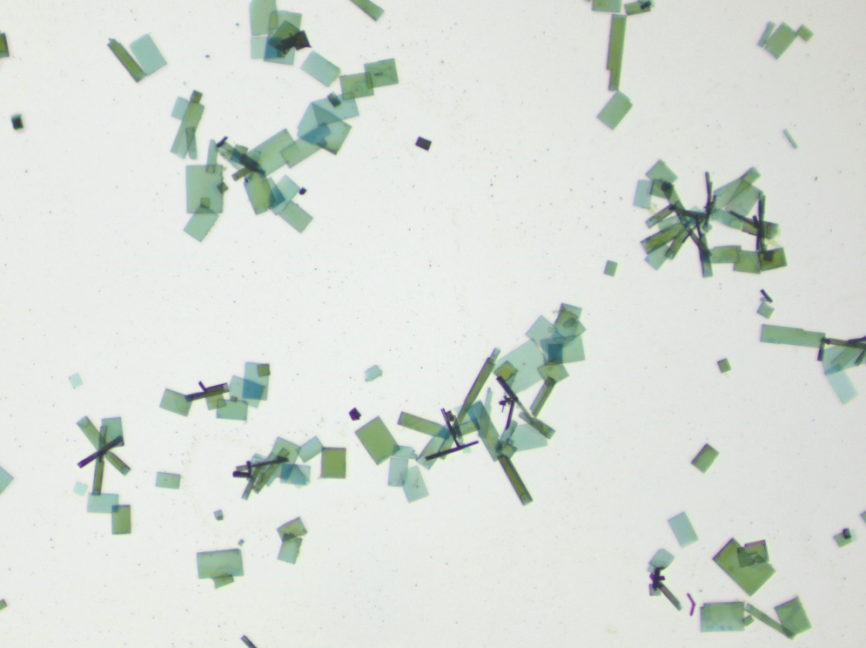


# Figure S7. Image of DTT-TCNQ micro-sheets under microscope.


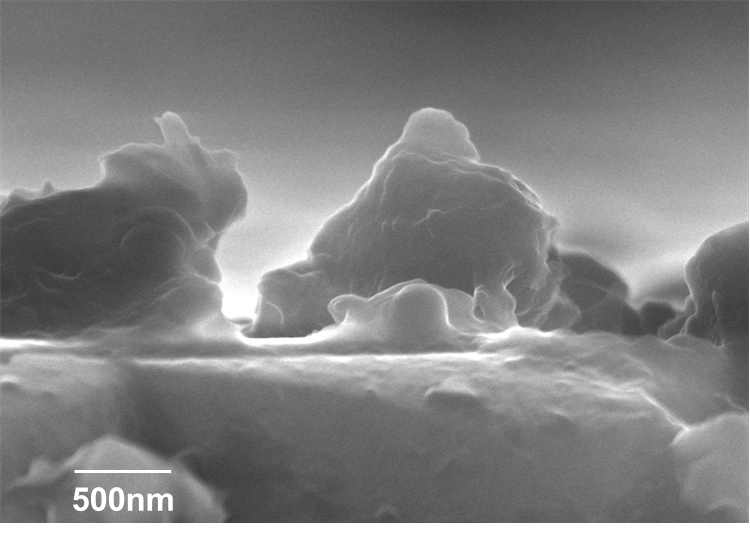


# Figure S8. Side view of DTT-TCNQ micro-sheets under SEM.


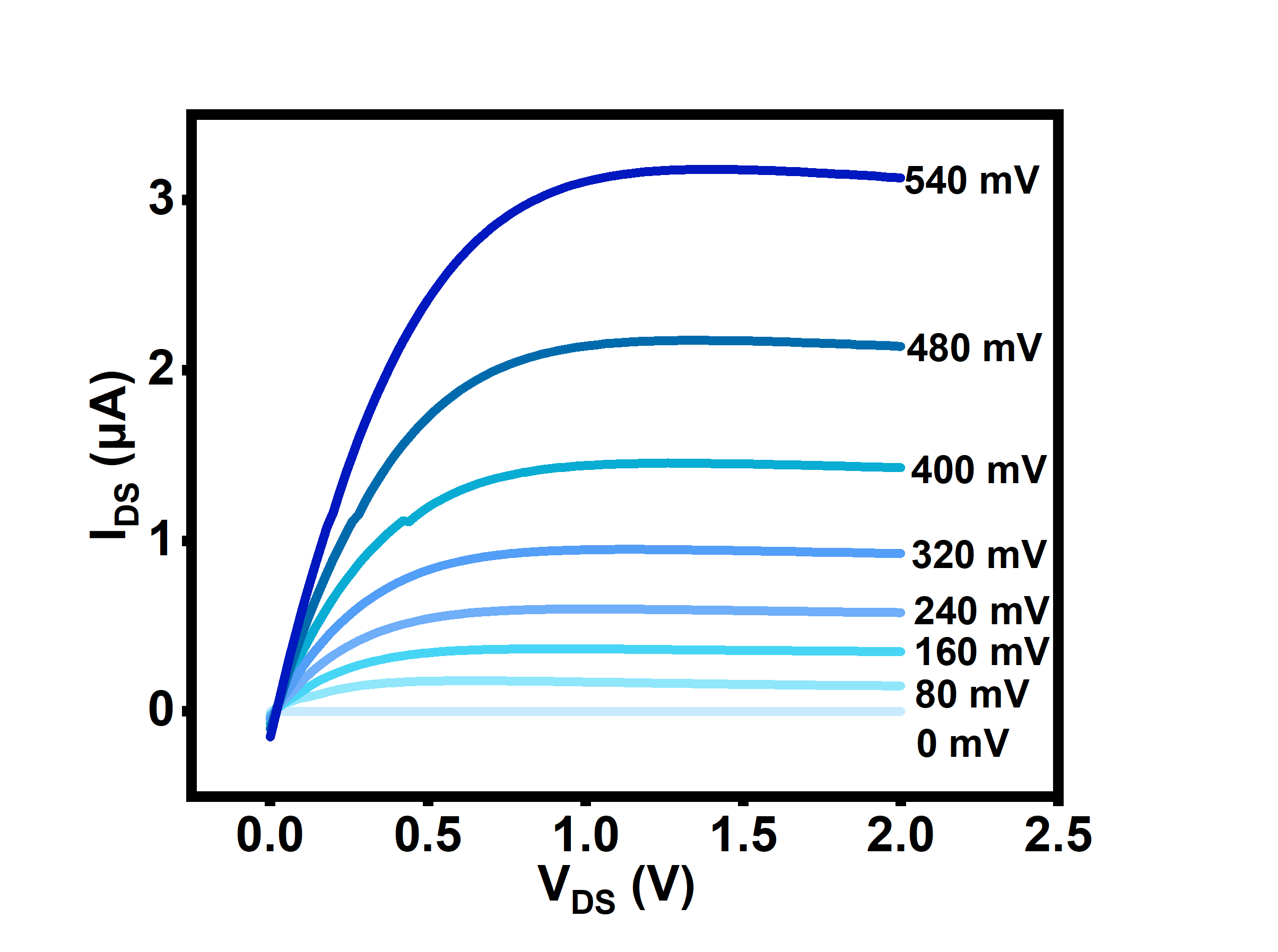


# Figure S9. I_DS_-V_DS_ curve of DTT-TCNQ enhanced memtransistor (V_GS_ ranging from 0 mV to 540 mV).

# Figure S10. (a) EPSC and (b) IPSC stimulated by different V_GS_ of electrical pulses (c) EPSC stimulated by different number, (d) widths of electrical pulses.


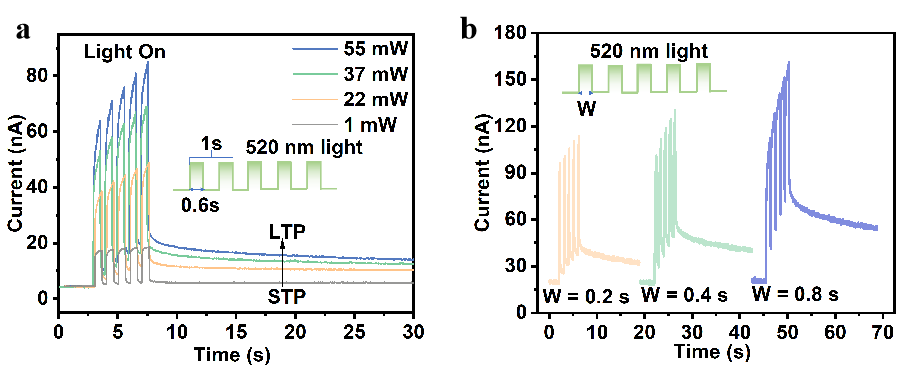


# Figure S11. EPSC stimulated by (a) varying power of 520 nm light pulses with a width of 0.6 s, (b) varying width of 520 nm light pulses .


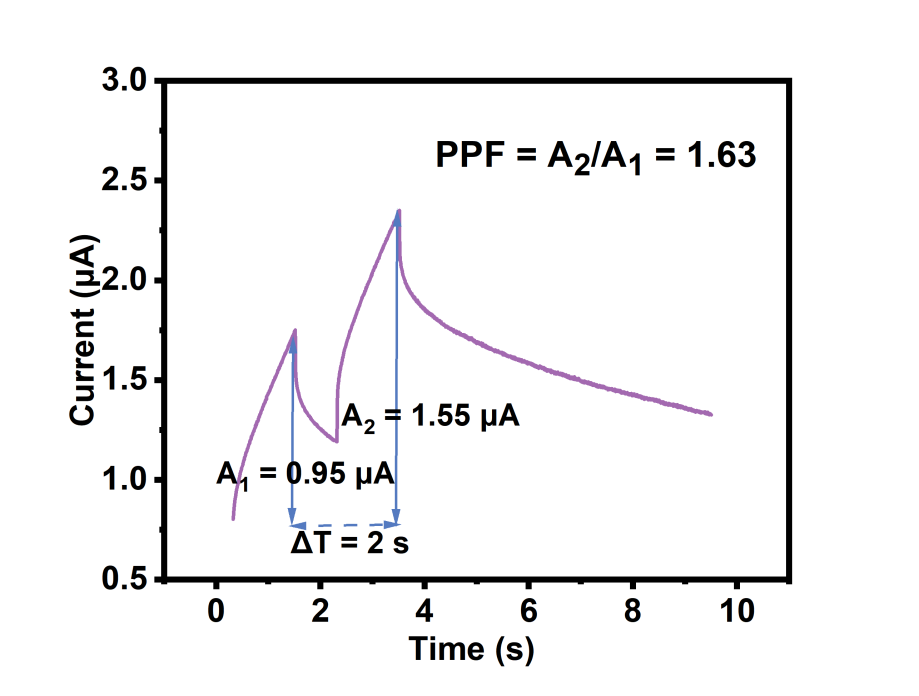

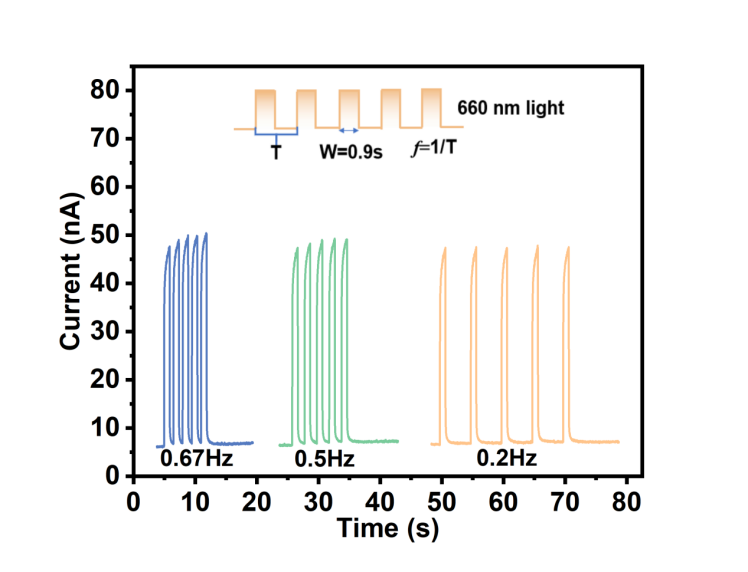


# Figure S12. Two optical pulses of the same intensity (395 nm, 60 mW).

# Figure S13. EPSC stimulated by varying frequencies of 660 nm optical pulses with a width of 0.9 s.

# Figure S14. Simulated “learning-forgetting-relearning” behavior under pulsed 450 nm light stimuli.


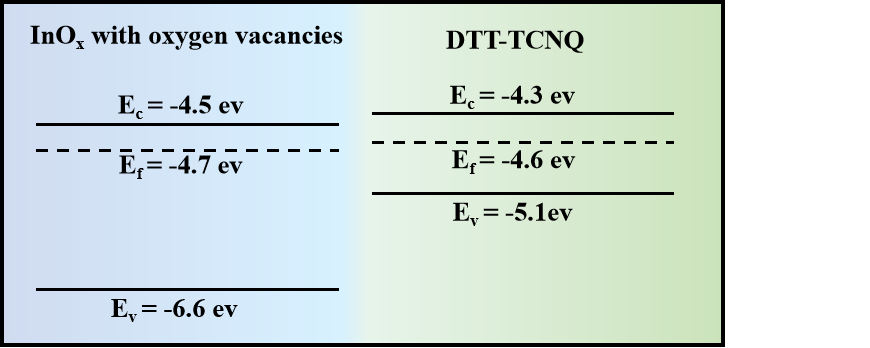


# Figure S15. Schematic energy band diagrams of InO_x_ and DTT-TCNQ before contact.


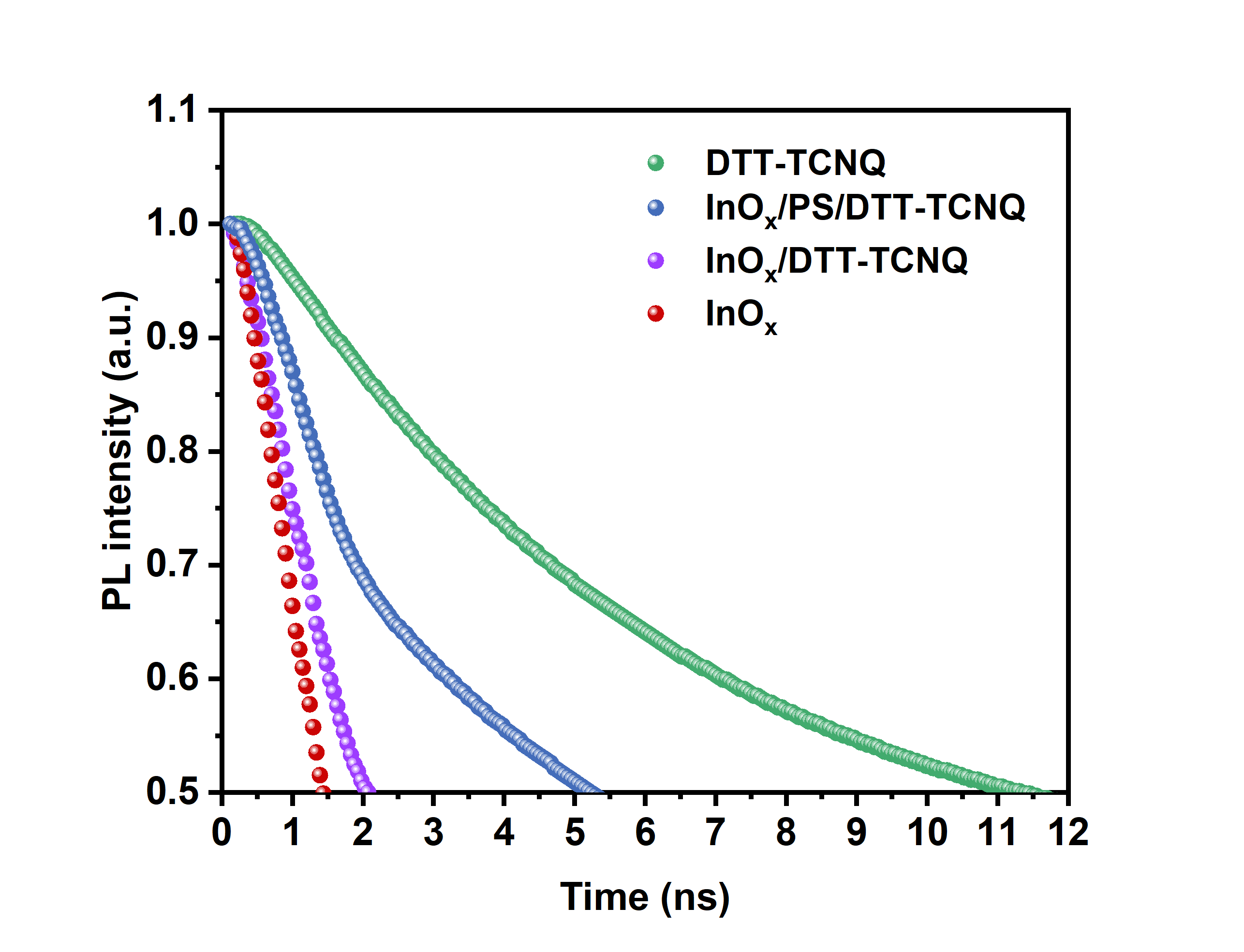


**Figure S16.** TRPL spectra of InO_x_, InO_x_/DTT-TCNQ and InO_x_/PS/DTT-TCNQ, DTT-TCNQ.

.

# Figure S17. (a) Schematic illustration of energy band alignment for InO_x_/DTT-TCNQ heterojunction under NIR light stimulation (V_GS_ > 0) and (b) under visible light stimulation (V_GS_ > 0).


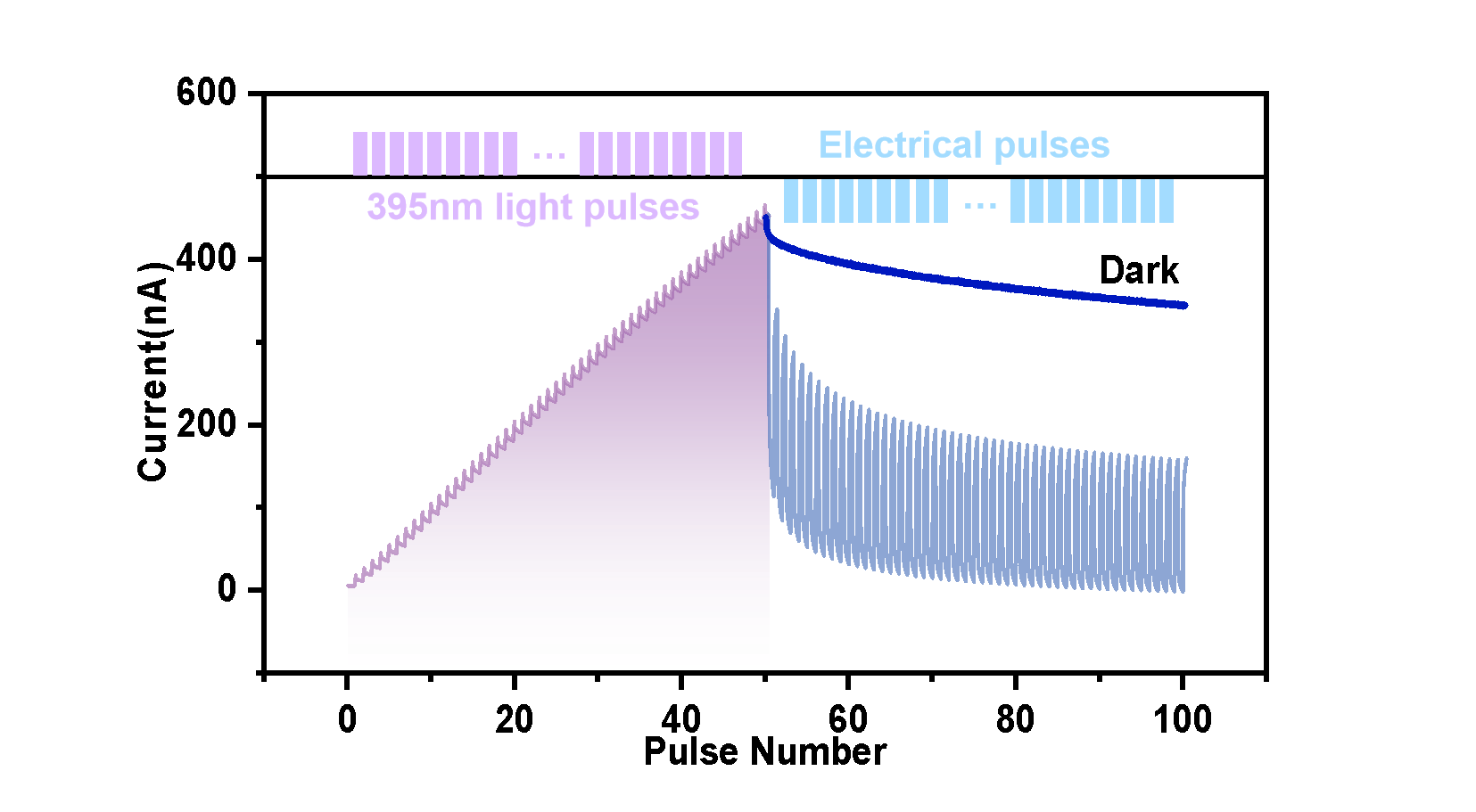


# Figure S18. Writing characteristics under 395nm light pulses and erasing characteristics under negative electrical pulses (-1 V).


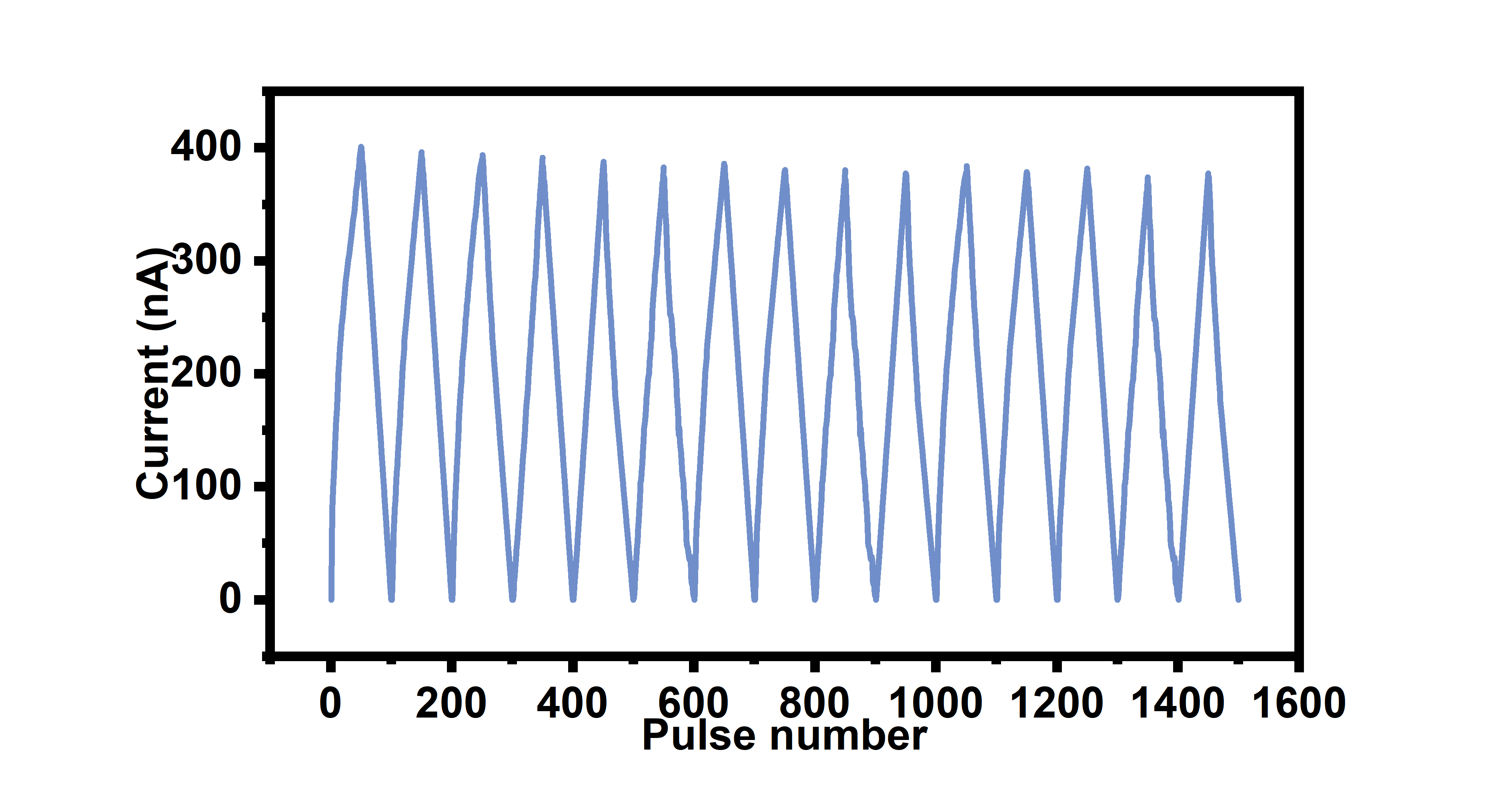


**Figure S19.** Synaptic current changes showing high reliability (50 times 395 nm, 2 mW pulses, 50 times 808 nm, 55 mW pulses, repeated 15 cycles)

# .


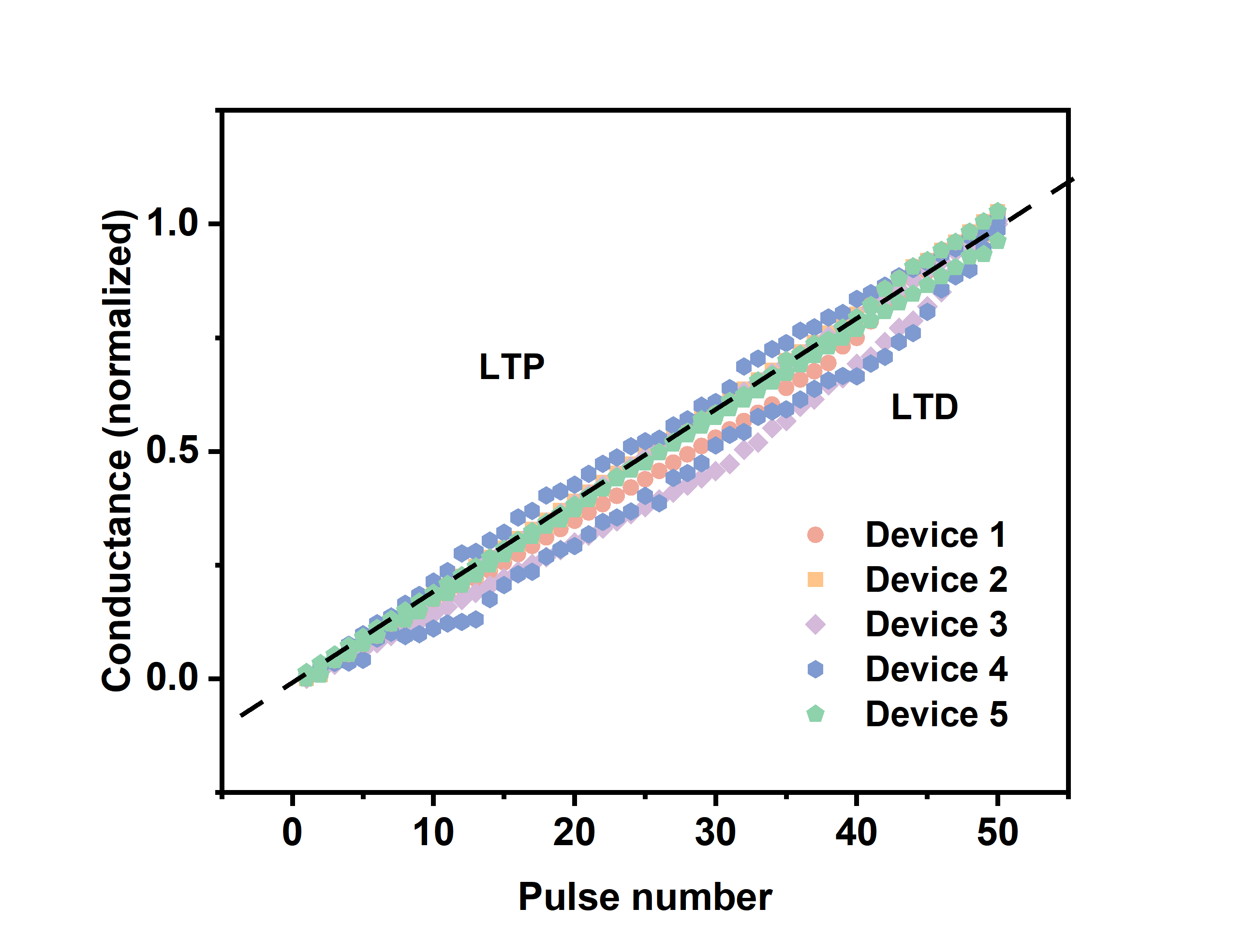


**Figure S20.** All devices were tested optical LTP with 395 nm light and optical LTD with 808 nm light.

# .


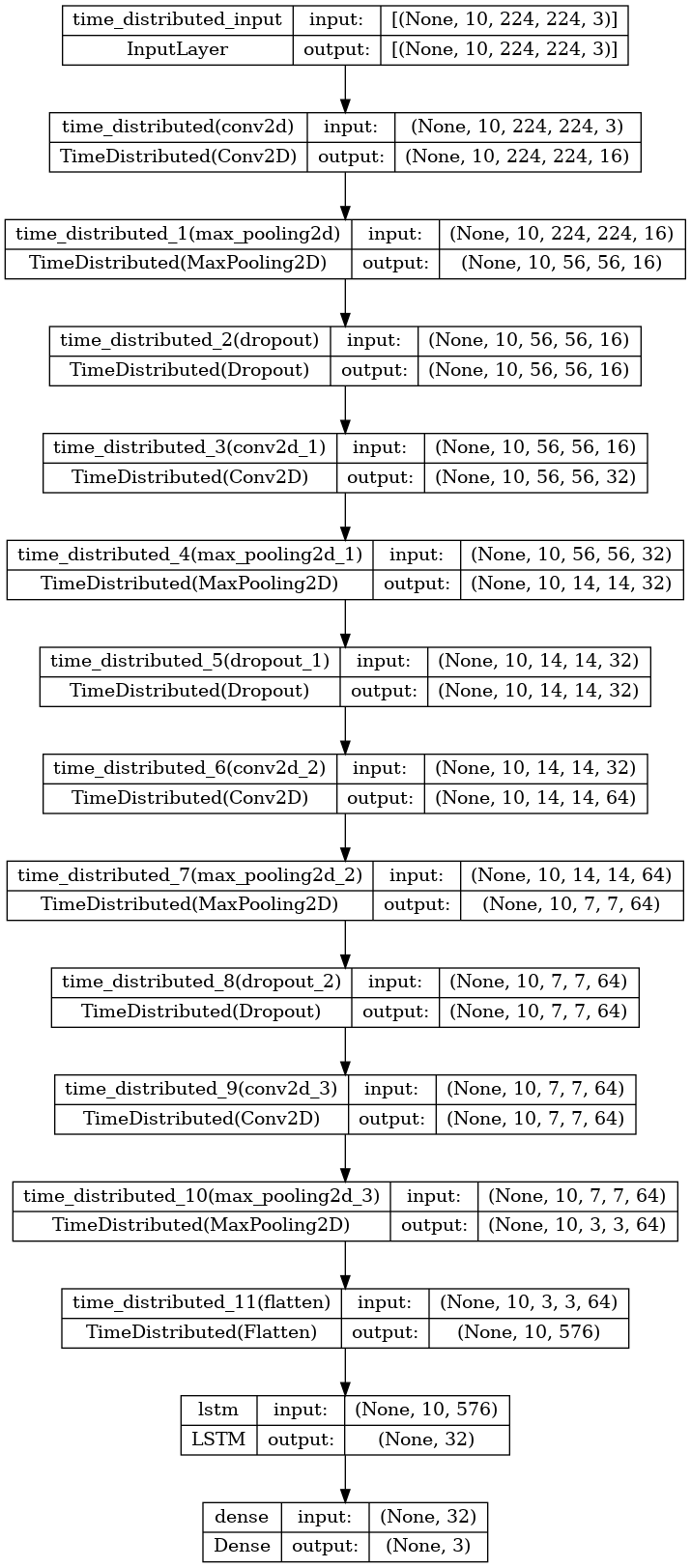


# Figure S21. Pixel images generated by the array for the letter I under UV light (λ = 395 nm) and green light (λ = 520 nm).

# Figure S22. Time-Distributed CNN-LSTM Network Architectures.


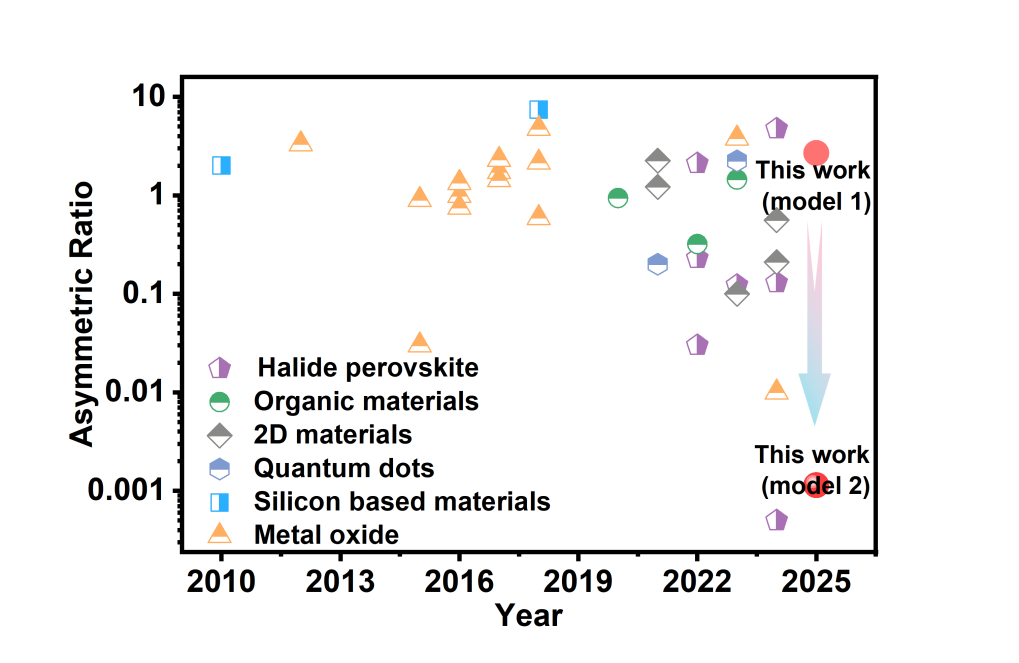

# Figure S23. Comparison of the AR characteristics of among different types of synaptic devices.

# Figure S24. Confusion matrix containing the confidences of each class for (a) model 1 and (b) model 2.

# Figure S25. Video targets under noise of varying brightness and varying sequence.


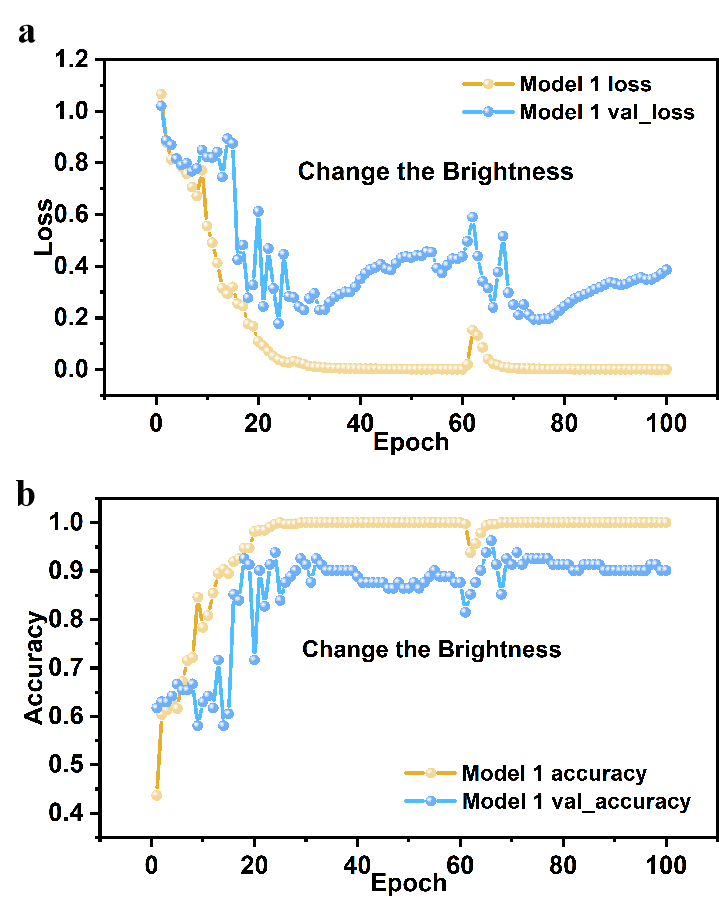


# Figure S26. (a) Loss and (b) accuracy of model 1 after adding the noise of varying brightness.


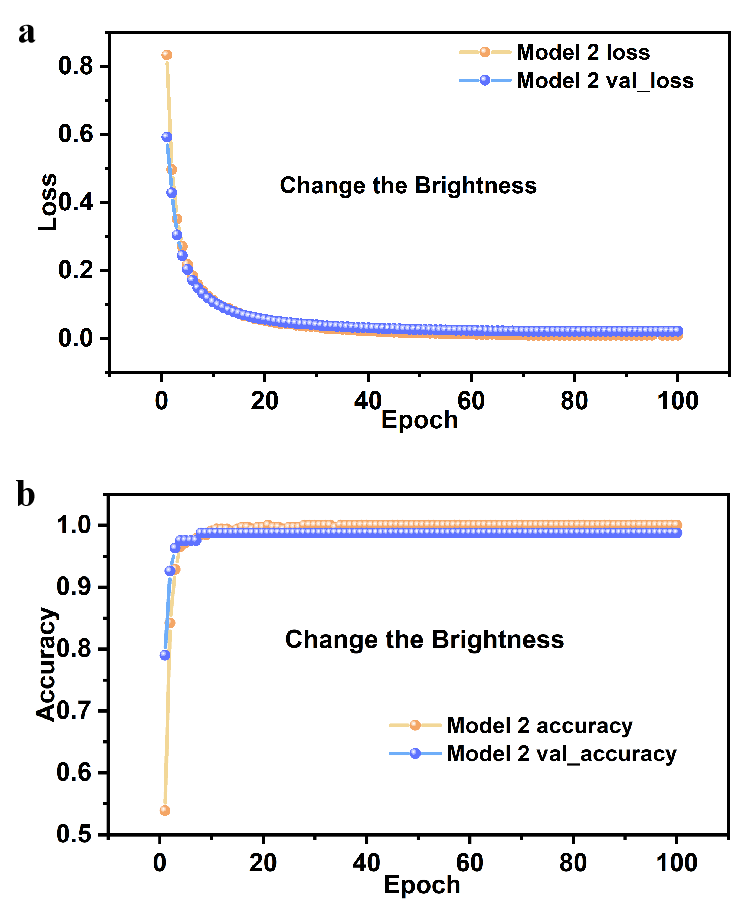


# Figure S27. (a) Loss and (b) accuracy of model 2 after adding the noise of varying brightness.


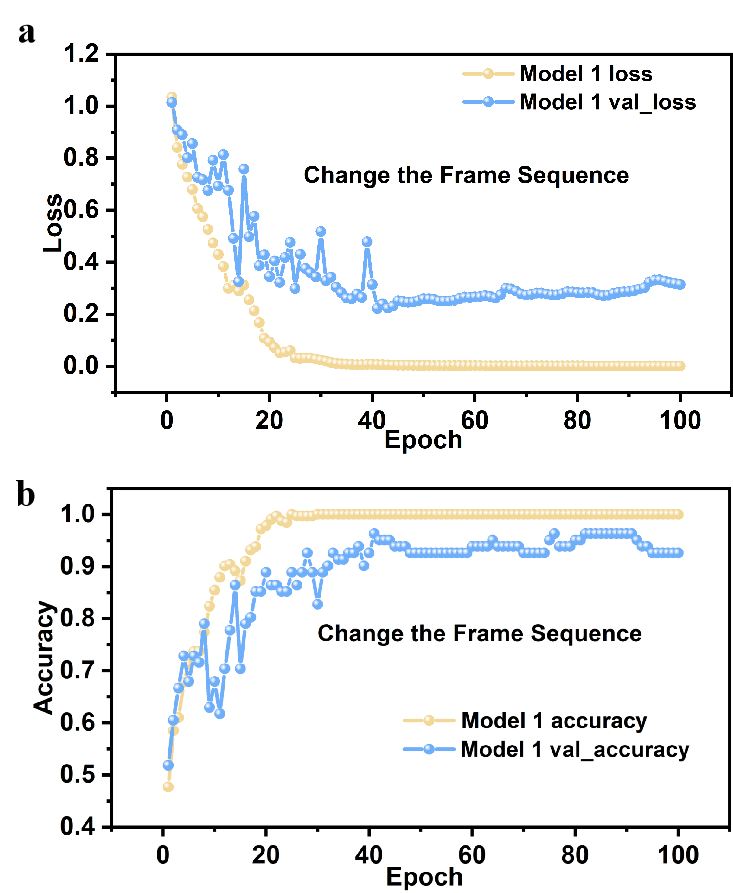


# Figure S28. (a) Loss and (b) accuracy of model 1 after adding the noise of varying frame sequence.


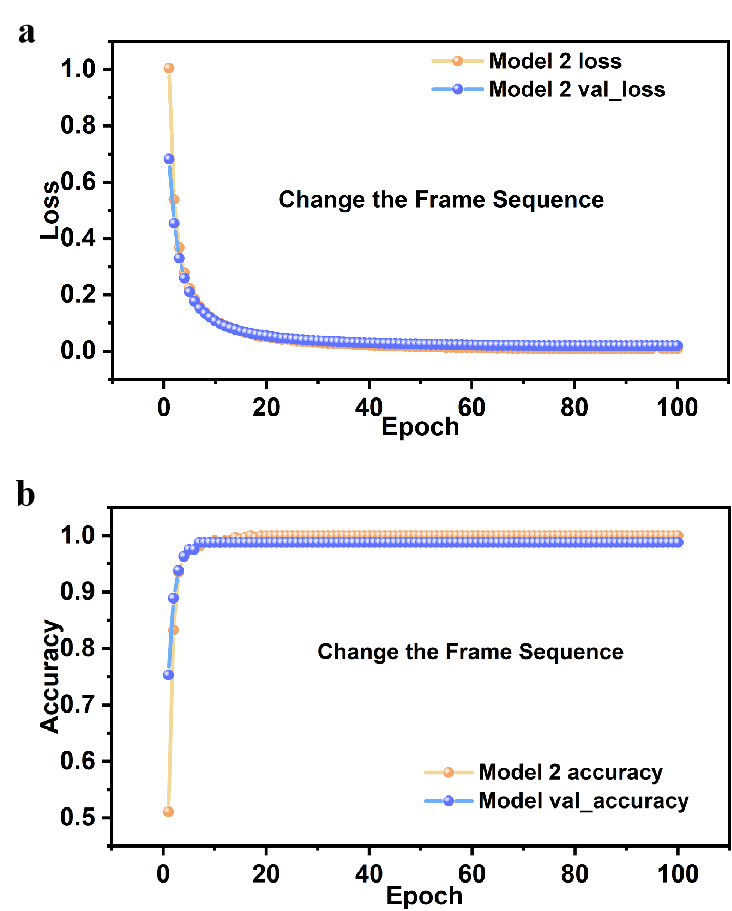


# Figure S29. (a) Loss and (b) accuracy of model 2 after adding the noise of varying frame sequence.


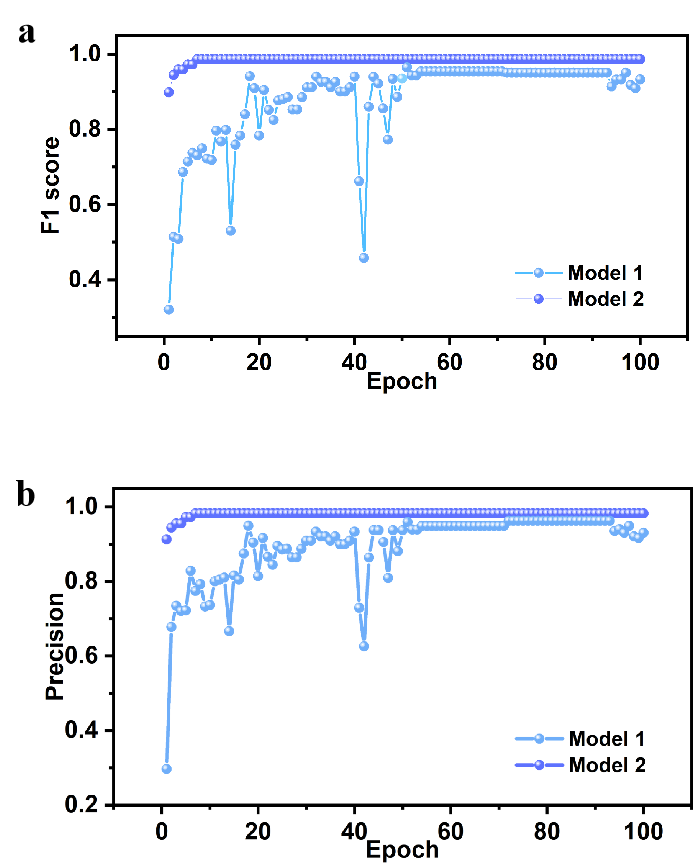


# Figure S30. (a) F1 score and (b) precision of model 1 and model 2.


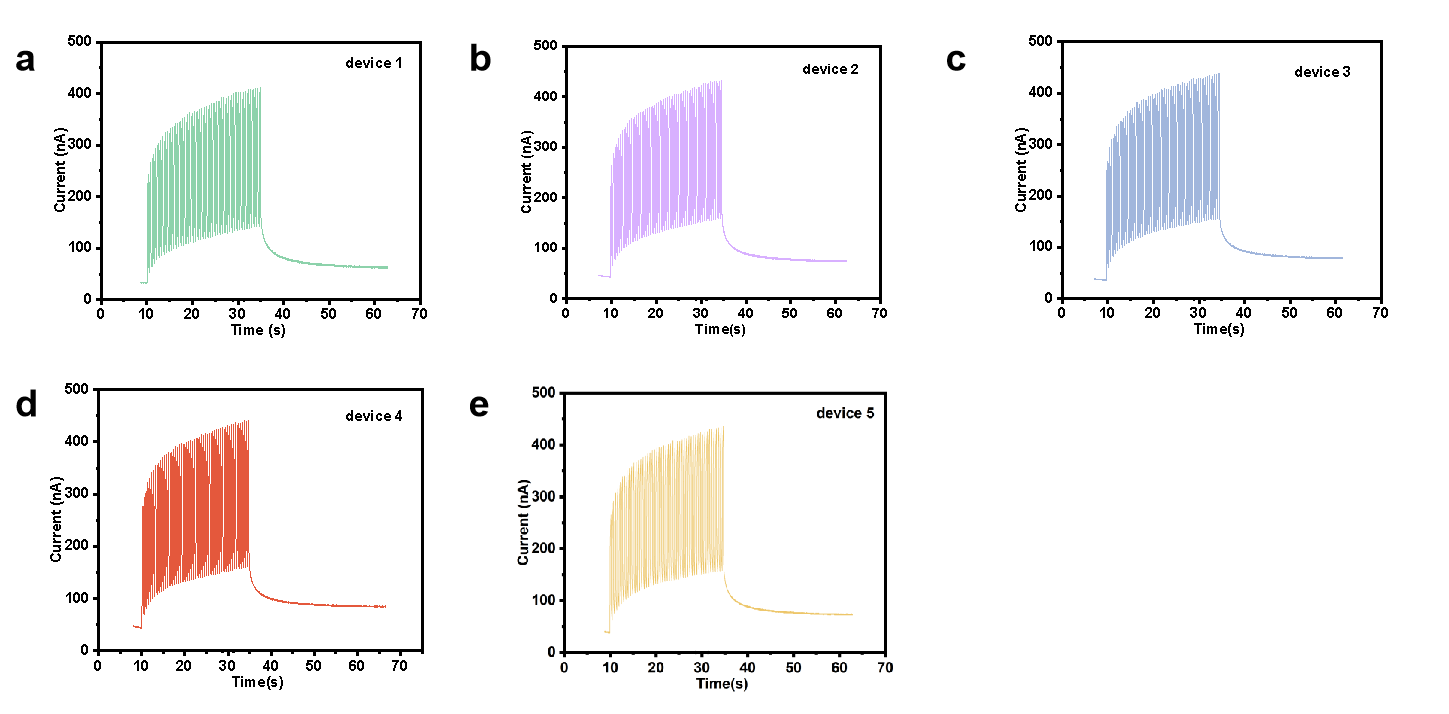


# Figure S31. The EPSC responses under 450 nm-43 mW light pulse for five devices.

**Table S1**. Comparison of the AR and NL characteristics of synaptic devices.

|  | **Material** | **Stimulus Source** | | **Asymmetric Ratio** | **Nonlinearity** | | **Reference** |
| --- | --- | --- | --- | --- | --- | --- | --- |
|  |  | **EPSC** | **IPSC** |  | ***α_p_*** | ***α_d_*** |  |
| Halide perovskite | OHP/PEN/ITO | electric and light pulses | -1 V | 0.1228 | 0.007 | 0.1298 | ^1^ |
|  | Al/V_2_O_5_–y/PVK/Cs_1–x_FAxPbBr_3_/b-PEI/ZnO/ITO | +5 V | -17 V | 4.75 | 5.52 | 0.77 | ^2^ |
|  | Au  /DJ-V-HP-BDAMA_2_Pb_3_I_10_/ Au | 1.5 V | -1.5 V | 5×10^-4^ | 0.002 | 0.0015 | ^3^ |
|  | RP-V-HP BDAMA_2_Pb_3_I_10_ | 1.5 V | -1.5 V | 0.13 | 0.09 | 0.22 | ^3^ |
|  | FABi_3_I_10_-FA_3_Bi_2_I_9_ | -0.6 V | 0.2 V | 2.11 | 0.52 | 2.63 | ^4^ |
|  | Au/CsCu_2_I_3_/ITO | -2 V | 2 V | 0.23 | 0.36 | 0.13 | ^5^ |
|  | Au/PEA_2_MA_4_Pb_5_I_16_/ITO | 1 V | -1 V | 0.03 | 0.07 | 0.1 | ^6^ |
| 2D materials | MoS2/h-BN | 1 V | 1V | 0 | 1.9 | 1.9 | ^7^ |
|  | α-In_2_Se_3_/CIPS | 785 nm | 2 V | 0.1 | 1.3 | 1.2 | ^8^ |
|  | HfS_2_ | 405 nm | 0.15-15 V | 2.25 | 2.3 | 0.05 | ^9^ |
|  | BP | 280 nm | 365 nm | 0 | 1 | 1 | ^10^ |
|  | ZTO/TiO_2_/Ti_3_C_2_T_x_ MXenes/SiO_2_ | 365 nm | 5V | 1.22 | 0.51 | 1.73 | ^11^ |
|  | VO-MXene/SiO_2_ | - | - | 0.21 | 1.43 | 1.64 | ^12^ |
|  | SnSe | 430 nm | 283 nm | 0.56 | 2.27 | 2.83 | ^13^ |
| Quantum dots | Pyr-GDY/Gr/PbS QDs | 980 nm | 450 nm | 1.7 | 1.9 | 0.2 | ^14^ |
|  | Gr/TiO_2_ QDs | 635 nm | 365 nm | 2.5 | 0.26 | 2.24 | ^15^ |
| Silicon-based | Cr/ Ag:a-Si | 3.2 V | -2.8 V | 2.01 | 2.37 | 4.38 | ^16^ |
|  | Ag / SiGe / p-Si | 5 V | 3 V | 7.43 | 10.7 | 3.27 | ^17^ |
| Metal oxide | Si-doped  β-Ga_2_O_3_/ZnO | 255 nm | 370 nm | 0.01 | 0.05 | 0.04 | ^18^ |
|  | Sn-doped β-Ga_2_O_3_ | 50 V | 10 V | 3.79 | 0.1 | 3.89 | ^19^ |
|  | TiN / Ti / AlO_x_ / TiN | 1.4 V | -1.6 V | 3.31 | 0.01 | 3.32 | ^20^ |
|  | Ti / AlO_x_ / TaO_x_ / Pt | 0.88 V | -1.15 V | 2.15 | 6.99 | 4.84 | ^21^ |
|  | TiN / ETML / HfO_x_ / TiN | 1.6 V | -1.5 V | 0.59 | 0.04 | 0.63 | ^22^ |
|  | Ta / Ta_2_O_5_ / Pd | 1.1 V | -1.4 V | 1.73 | 7.77 | 6.04 | ^23^ |
|  | TiN / HfO_2_ / Pt | 1.1 V | -0.8 V | 1.43 | 8.57 | 10 | ^24^ |
|  | TiN / TEL / HfO_x_ / TiN | 1.7 V | -1.5 V | 2.3 | 0.96 | 3.26 | ^25^ |
|  | Pt / TiO_x_ / Pt | 3 V | - 3 V | 0.99 | 3.51 | 4.5 | ^26^ |
|  | W / TaO_y_ / Ta_2_O_5-x_ / AlO_y_ / Al | 7 V | -7 V | 1.34 | 4.3 | 2.96 | ^27^ |
|  | Al / AlO_x_ / HfO_2_ / Ti / TiN | 0.9 V | -1 V | 0.75 | 2.03 | 1.28 | ^28^ |
|  | Ta / TaO_x_ / TiO_2_ / Ti | 3 V | -3 V | 0.03 | 0.66 | 0.69 | ^29^ |
|  | TiN / PCMO / Pt | -1.5 V, -3.5 V | -0.5 V, -2.5 V | 0.9 | 3.4 | 2.5 | ^30^ |
|  | Pt / STO / Nb-STO | 1.62 V | -1.8 V | 4.74 | 3.6 | 8.34 | ^31^ |
| Organic Materials | DPP-DTT/AlO_x_/  PVDF-AFP | 365 nm | 5 V | 0.68 | 1.62 | 0.94 | ^32^ |
|  | BTBTT6-syn | 370 nm | -15 V | 1×10^-4^ | 0.2903 | 0.2902 | ^33^ |
|  | Y_6_/Al_2_O_3_/In_2_O_3_ | 365 nm | 800 nm | 1.65 | 0.2 | 1.45 | ^34^ |
|  | Si NCs/P3HT | 532 nm | 375 nm | 2.11 | 1.79 | 0.32 | ^35^ |
|  | **DTT-TCNQ/InO_x_/ZrO_x_** | **395nm** | **-0.5V** | **2.691** | **0.00191** | **2.6933** | **This work** |
|  |  | **395nm** | **808nm** | **0.00114** | **0.00191** | **0.00305** | **This work** |

# References

1. H. Wei, G. Yao, Y. Ni, L. Yang, J. Liu, L. Sun, X. Zhang, J. Yang, Y. Xiao, F. Zheng, W. Xu, Adv. Funct. Mater. 2023, 33, 2304000;
2. Y. R. Park, G. Wang, Adv. Funct. Mater. 2023, 34;
3. S. J. Kim, I. H. Im, J. H. Baek, S. Choi, S. H. Park, D. E. Lee, J. Y. Kim, S. Y. Kim, N.-G. Park, D. Lee, J. J. Yang, H. W. Jang, Nat. Nanotechnol. 2024;
4. R. Yang, H.-M. Huang, Q.-H. Hong, X.-B. Yin, Z.-H. Tan, T. Shi, Y.-X. Zhou, X.-S. Miao, X.-P. Wang, S.-B. Mi, C.-L. Jia, X. Guo, Adv. Funct. Mater. 2018, 28, 1704455;
5. K. J. Kwak, J. H. Baek, D. E. Lee, I. h. Im, J. Kim, S. J. Kim, Y. J. Lee, J. Y. Kim, H. W. Jang, Nano Lett. 2022, 22, 6010;
6. S. J. Kim, T. H. Lee, J.-M. Yang, J. W. Yang, Y. J. Lee, M.-J. Choi, S. A. Lee, J. M. Suh, K. J. Kwak, J. H. Baek, I. H. Im, D. E. Lee, J. Y. Kim, J. Kim, J. S. Han, S. Y. Kim, D. Lee, N.-G. Park, H. W. Jang, Mater. Today 2022, 52, 19;
7. S. Seo, B.-S. Kang, J.-J. Lee, H.-J. Ryu, S. Kim, H. Kim, S. Oh, J. Shim, K. Heo, S. Oh, J.-H. Park, Nat. Commun. 2020, 11, 3936;
8. B. Das, S. Baek, J. Niu, C. Jang, Y. Lee, S. Lee, ACS Nano 2023, 17, 21297;
9. H. Xiong, L. Xu, C. Gao, Q. Zhang, M. Deng, Q. Wang, J. Zhang, D. Fuchs, W. Li, A. Cui, L. Shang, K. Jiang, Z. Hu, J. Chu, ACS Appl. Mater. Interfaces 2021, 13, 50132;
10. T. Ahmed, M. Tahir, M. X. Low, Y. Ren, S. A. Tawfik, E. L. H. Mayes, S. Kuriakose, S. Nawaz, M. J. S. Spencer, H. Chen, M. Bhaskaran, S. Sriram, S. Walia, Adv. Mater. 2021, 33, 2004207;
11. T. Zhao, C. Zhao, W. Xu, Y. Liu, H. Gao, I. Z. Mitrovic, E. G. Lim, L. Yang, C. Z. Zhao, Adv. Funct. Mater. 2021, 31, 2106000;
12. D. Tan, Z. Zhang, H. Shi, N. Sun, Q. Li, S. Bi, J. Huang, Y. Liu, Q. Guo, C. Jiang, Adv. Mater. 2024, 36, 2407751;
13. Z. Liu, Y. Wang, Y. Zhang, S. Sun, T. Zhang, Y.-J. Zeng, L. Hu, F. Zhuge, B. Lu, X. Pan, Z. Ye, Adv. Mater. 2024, n/a, 2410783;
14. Y.-X. Hou, Y. Li, Z.-C. Zhang, J.-Q. Li, D.-H. Qi, X.-D. Chen, J.-J. Wang, B.-W. Yao, M.-X. Yu, T.-B. Lu, J. Zhang, ACS Nano 2021, 15, 1497;
15. J. Liang, X. Yu, J. Qiu, M. Wang, C. Cheng, B. Huang, H. Zhang, R. Chen, W. Pei, H. Chen, ACS Appl. Mater. Interfaces 2023, 15, 9584;
16. S. H. Jo, T. Chang, I. Ebong, B. B. Bhadviya, P. Mazumder, W. Lu, Nano Lett. 2010, 10, 1297;
17. S. Choi, S. H. Tan, Z. Li, Y. Kim, C. Choi, P.-Y. Chen, H. Yeon, S. Yu, J. Kim, Nat. Mater. 2018, 17, 335;
18. S. Sun, T. Zhang, S. Jin, X. Pan, J. Lu, Z. Ye, B. Lu, Adv. Funct. Mater. 2024, 34, 2401403;
19. Y. Yoon, Y. Kim, W. S. Hwang, M. Shin, Adv. Electron. Mater. 2023, 9, 2300098;
20. Y. Wu, S. Yu, H.-S. P. Wong, Y.-S. Chen, H.-Y. Lee, S.-M. Wang, P. Gu, F. T. Chen, M.-J. Tsai, 2012 4th IEEE International Memory Workshop 2012, 1;
21. Y. Sun, H. Xu, C. Wang, B. Song, H. Liu, Q. Liu, S. Liu, Q. Li, IEEE Electron Device Lett. 2018, 39, 1298;
22. W. Wu, H. Wu, B. Gao, P. Yao, X. Zhang, X. Peng, S. Yu, H. Qian, 2018 IEEE Symposium on VLSI Technology 2018, 103;
23. S. Choi, J. H. Shin, J. Lee, P. Sheridan, W. D. Lu, Nano Lett. 2017, 17, 3113;
24. E. Covi, S. Brivio, J. Frascaroli, M. Fanciulli, S. Spiga, ECS Trans. 2017, 75, 85;
25. W. Wu, H. Wu, B. Gao, N. Deng, S. Yu, H. Qian, IEEE Electron Device Lett. 2017, 38, 1019;
26. A. Serb, J. Bill, A. Khiat, R. Berdan, R. Legenstein, T. Prodromakis, Nat. Commun. 2016, 7, 12611;
27. X. Li, H. Wu, B. Gao, W. Wu, D. Wu, N. Deng, J. Cai, H. Qian, Nanotechnology 2016, 27;
28. J. Woo, K. Moon, J. Song, S. Lee, M. Kwak, J. Park, H. Hwang, IEEE Electron Device Lett. 2016, 37, 994;
29. L. Gao, I.-T. Wang, P.-Y. Chen, S. B. K. Vrudhula, J.-s. Seo, Y. Cao, T. H. Hou, S. Yu, Nanotechnology 2015, 26;
30. J. Jang, S. Park, G. W. Burr, H. Hwang, Y.-H. Jeong, IEEE Electron Device Lett. 2015, 36, 457;
31. J.-M. Yang, J.-H. Lee, Y.-K. Jung, S.-Y. Kim, J.-H. Kim, S.-G. Kim, J.-H. Kim, S. Seo, D.-A. Park, J.-W. Lee, A. Walsh, J.-H. Park, N.-G. Park, Adv. Sci. 2022, 9, 2200168;
32. X. Wang, S. Yang, Z. Qin, B. Hu, L. Bu, G. Lu, Adv. Mater. 2023, 35, 2303699;
33. T. Jiang, Y. Wang, Y. Zheng, L. Wang, X. He, L. Li, Y. Deng, H. Dong, H. Tian, Y. Geng, L. Xie, Y. Lei, H. Ling, D. Ji, W. Hu, Nat. Commun. 2023, 14, 2281;
34. D. Li, H. Ren, Y. Chen, Y. Tang, K. Liang, Y. Wang, F. Li, G. Liu, L. Meng, B. Zhu, Adv. Funct. Mater. 2023, 33, 2303198;
35. Y. Wang, Y. Zhu, Y. Li, Y. Zhang, D. Yang, X. Pi, Adv. Funct. Mater. 2022, 32, 2107973.
